# Supplementary material for: Continuous strain tuning of oxygen evolution catalysts with anisotropic thermal expansion
Source: Nat Commun. 2024 Feb 28;15:1780. doi: 10.1038/s41467-024-46216-9 (PMC10901830; doi:10.1038/s41467-024-46216-9)
Supplement: Supplementary file 1 — Supplementary Information [file 41467_2024_46216_MOESM1_ESM.pdf]

Supplementary Information

Continuous strain tuning of oxygen evolution catalysts with anisotropic thermal expansion

Yu Du[1], Fakang Xie[1], Mengfei Lu[1,2], Rongxian Lv[3], Wangxi Liu[1,2], Yuandong Yan[1], Shicheng Yan[1*], and Zhigang Zou[1,2]

[1]Collaborative Innovation Center of Advanced Microstructures, National Laboratory of Solid State Microstructures, Eco-materials and Renewable Energy Research Center (ERERC), College of Engineering and Applied Sciences, Nanjing University, No. 22 Hankou Road, Nanjing, Jiangsu 210093, P. R. China.
[2]Jiangsu Key Laboratory for Nano Technology, Nanjing University, No. 22 Hankou Road, Nanjing, Jiangsu 210093, P. R. China.
[3]Industrial Center, Nanjing Institute of Technology, No. 1 Hongjing Avenue, Nanjing, Jiangsu 211167, P. R. China.

*Correspondence: yscfei@nju.edu.cn

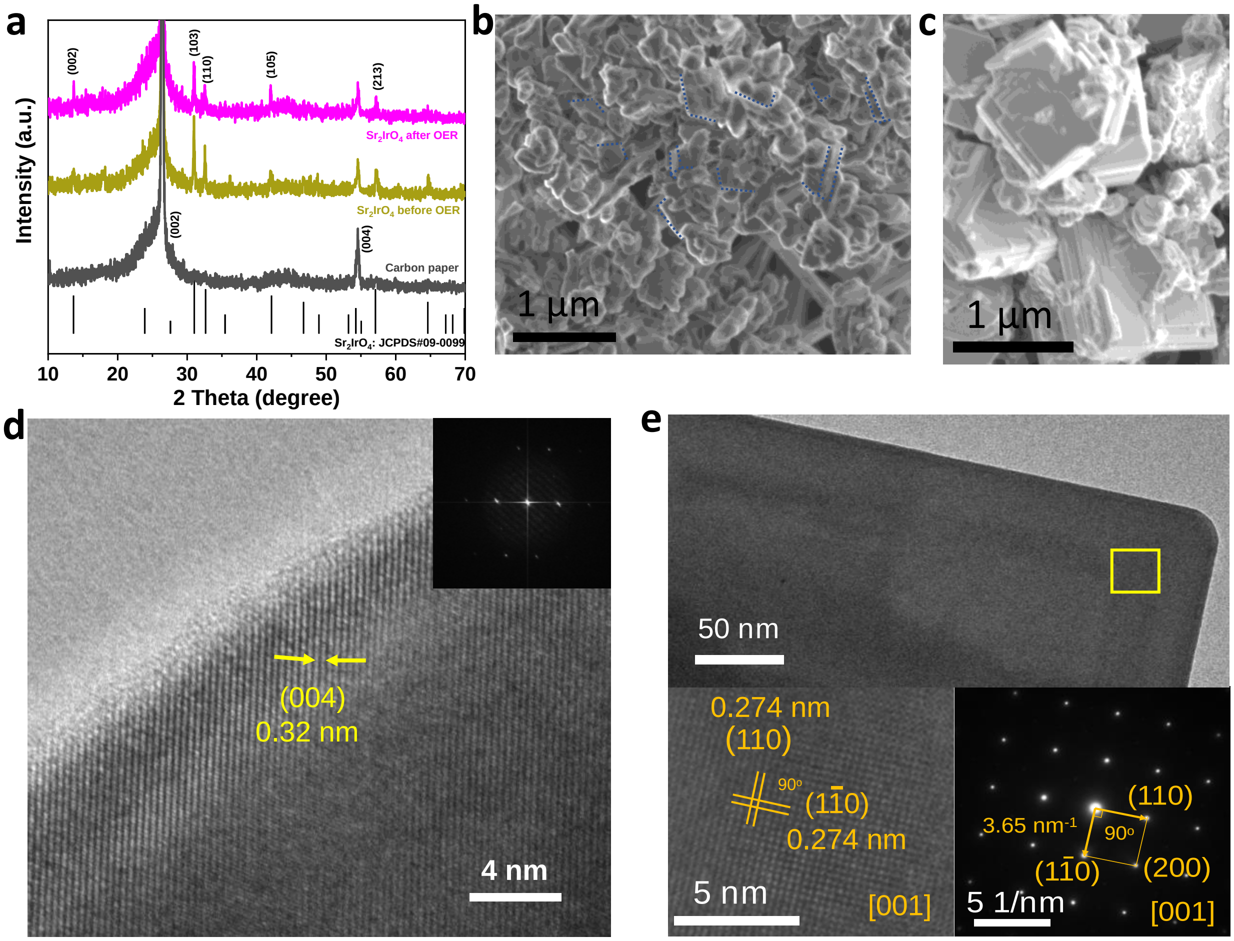

Supplementary Fig. 1 | a, The XRD patterns for Sr_2IrO_4 before/after OER and carbon paper substrate. The XRD peaks at 26.38^o and 54.54^o are assigned to the (002) and (004) crystal plane of graphitic carbon, respectively. **b,** SEM image for undeveloped Sr_2IrO_4 particles. The dotted lines trace the profile of the undeveloped Sr_2IrO_4 particles. **c,** SEM image for the relatively perfect Sr_2IrO_4 crystals formed by stacking (001) facet along the c-axis crystallographic direction. **d,** HRTEM lattice image for an undeveloped Sr_2IrO_4 single crystal with irregular shape. Inset shows the FFT image. **e,** TEM image, HRTEM lattice image, and SAED pattern for a relatively perfect Sr_2IrO_4 single crystal to show the possible growth direction and possible exposed facet. The HRTEM lattice image and SAED pattern were collected on the yellow box area.

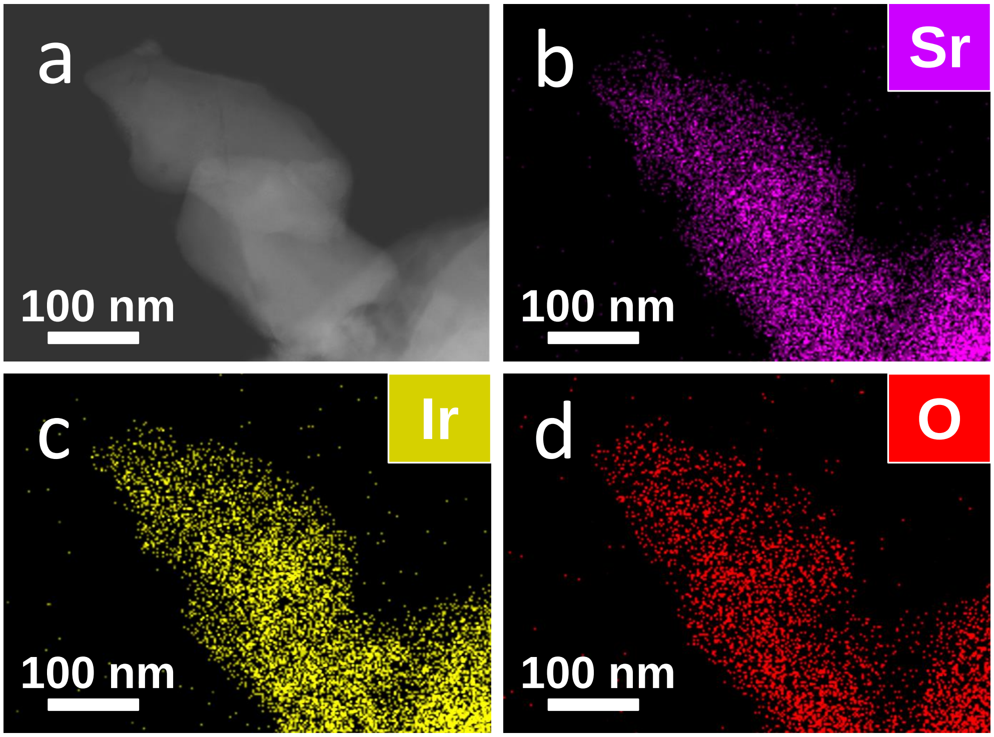

Supplementary Fig. 2 | a-d, HAADF-STEM image of the Sr_2IrO_4 particles (**a**), and Sr (**b**), Ir (**c**), and O (**d**) distributions in Sr_2IrO_4 particles.

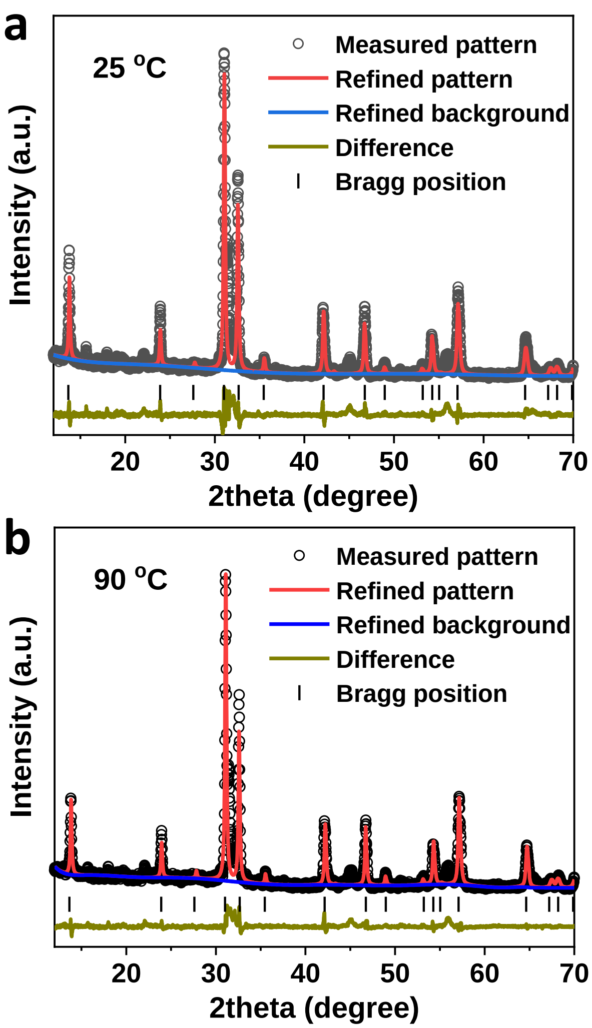

Supplementary Fig. 3 | a,b, Refined XRD patterns of Sr_2IrO_4 fitted by $I4_1/acd$ structure. Experimental patterns were measured under 25 ^{o}C **(a)** and 90 ^{o}C **(b)**.

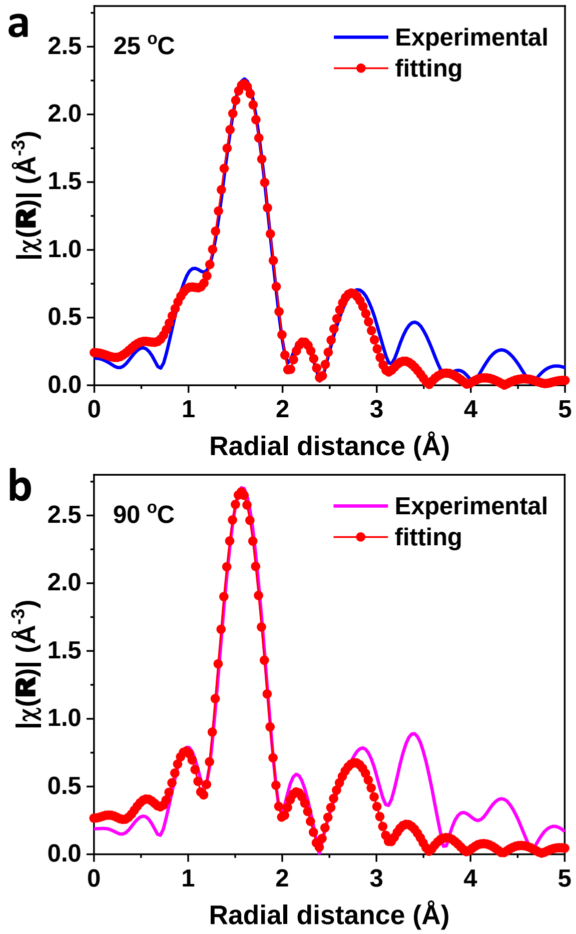

Supplementary Fig. 4 | a,b, EXAFS fitting curves for Ir L_3-edge of Sr_2IrO_4 under 25 °C **(a)** and 90 °C **(b)** (The plots were not corrected for phase shift).

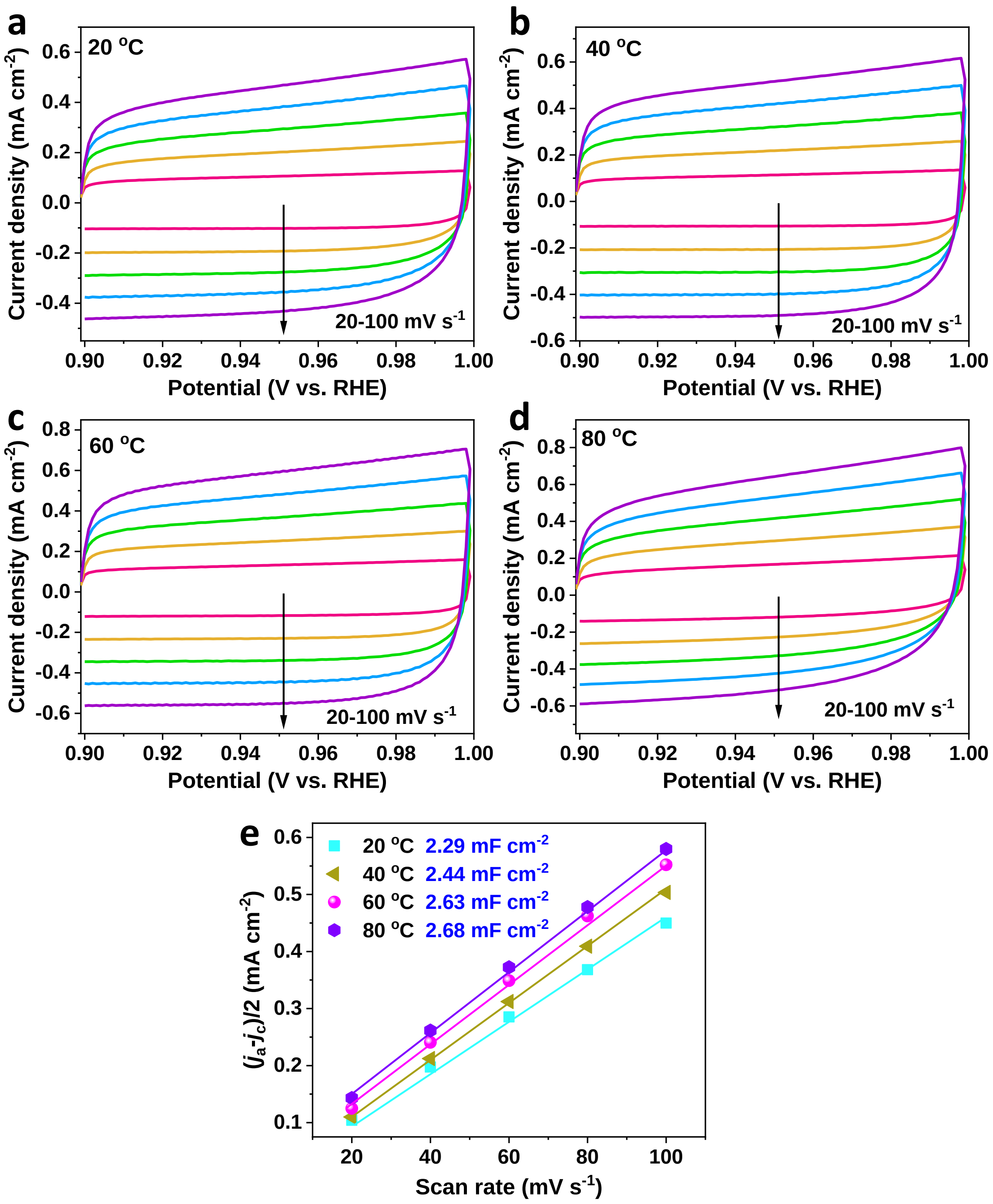

Supplementary Fig. 5 | a-d, CV curves of Sr_2IrO_4 with different scan rates in non-faradic region under 20 °C **(a)**, 40 °C **(b)**, 60 °C **(c)**, and 80 °C **(d)**. **e,** The non-faraday current density at 0.95 V as a function of scan rates of Sr_2IrO_4. The slope of fitting line is the double-layer capacitance.

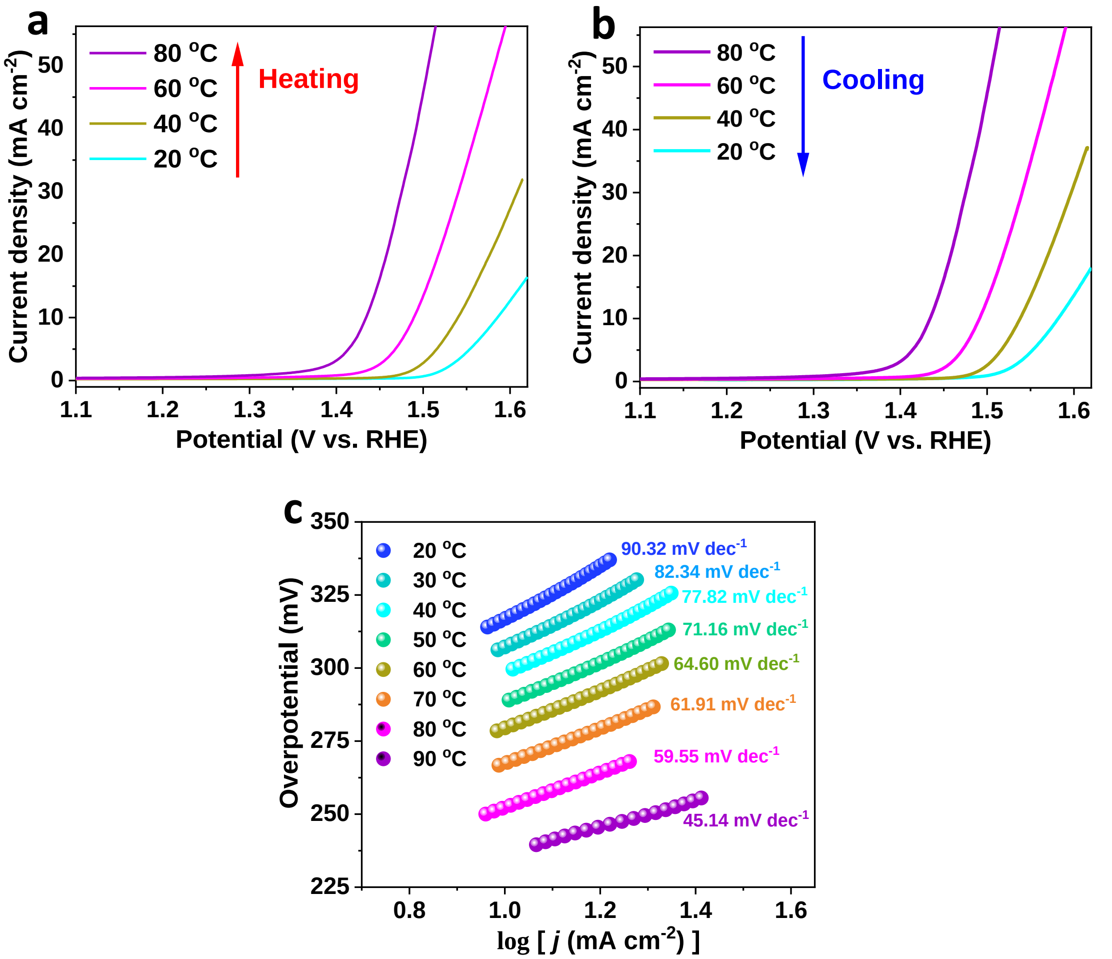

Supplementary Fig. 6 | a, LSV curves for Sr_2IrO_4 during heating process from 20 °C to 80 °C in 1.0 M KOH. **b,** LSV curves of Sr_2IrO_4 during cooling process from 20 °C to 80 °C in 1.0 M KOH. (scan rate, 5 mV/s; mass loading, 0.4 mg cm^{-2}; 90% iR-drop compensation is utilized; the pH of the electrolyte under different temperatures are shown in Supplementary Table 7.) **c,** Tafel slopes.

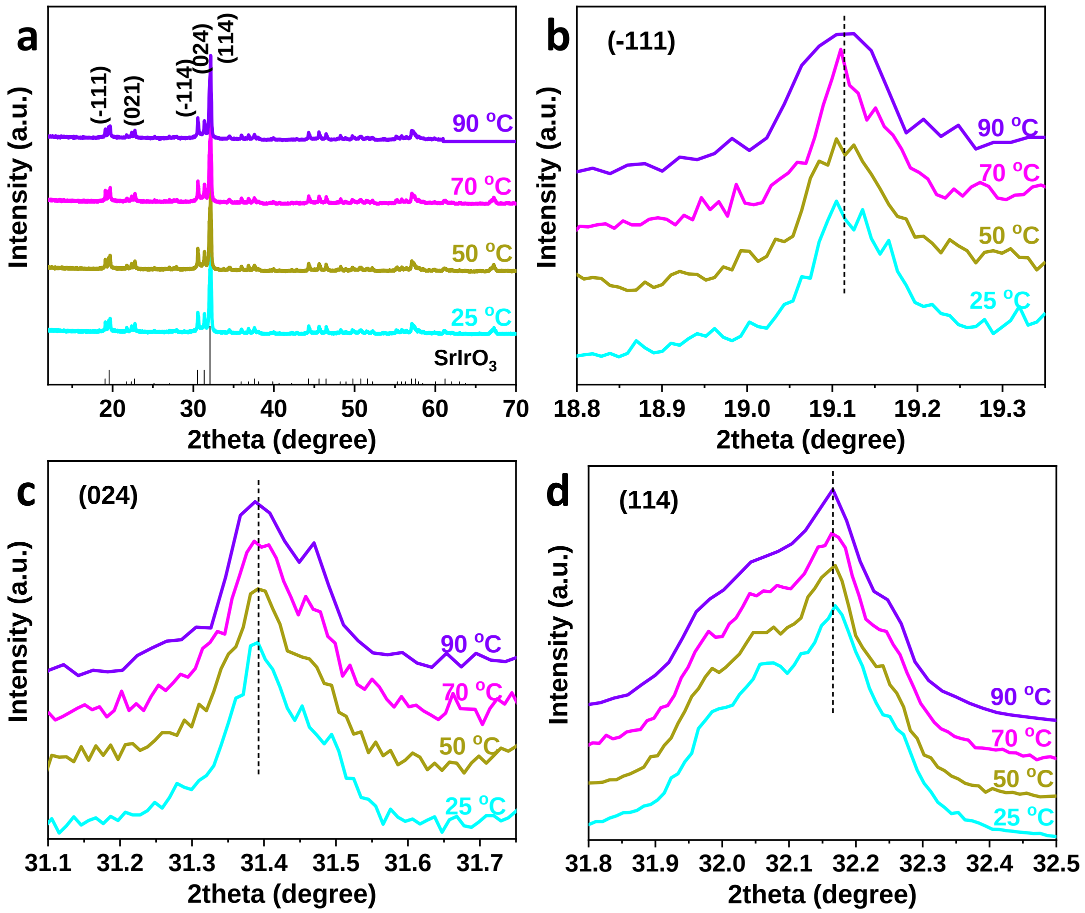

Supplementary Fig. 7 | a, XRD patterns for 6H-phase $SrIrO_3$ under different temperatures and the standard pattern of JCPDS-25-0897. **b-d,** Temperature dependence of (-111) **(b)**, (024) **(c)**, and (114) **(d)** diffraction peaks.

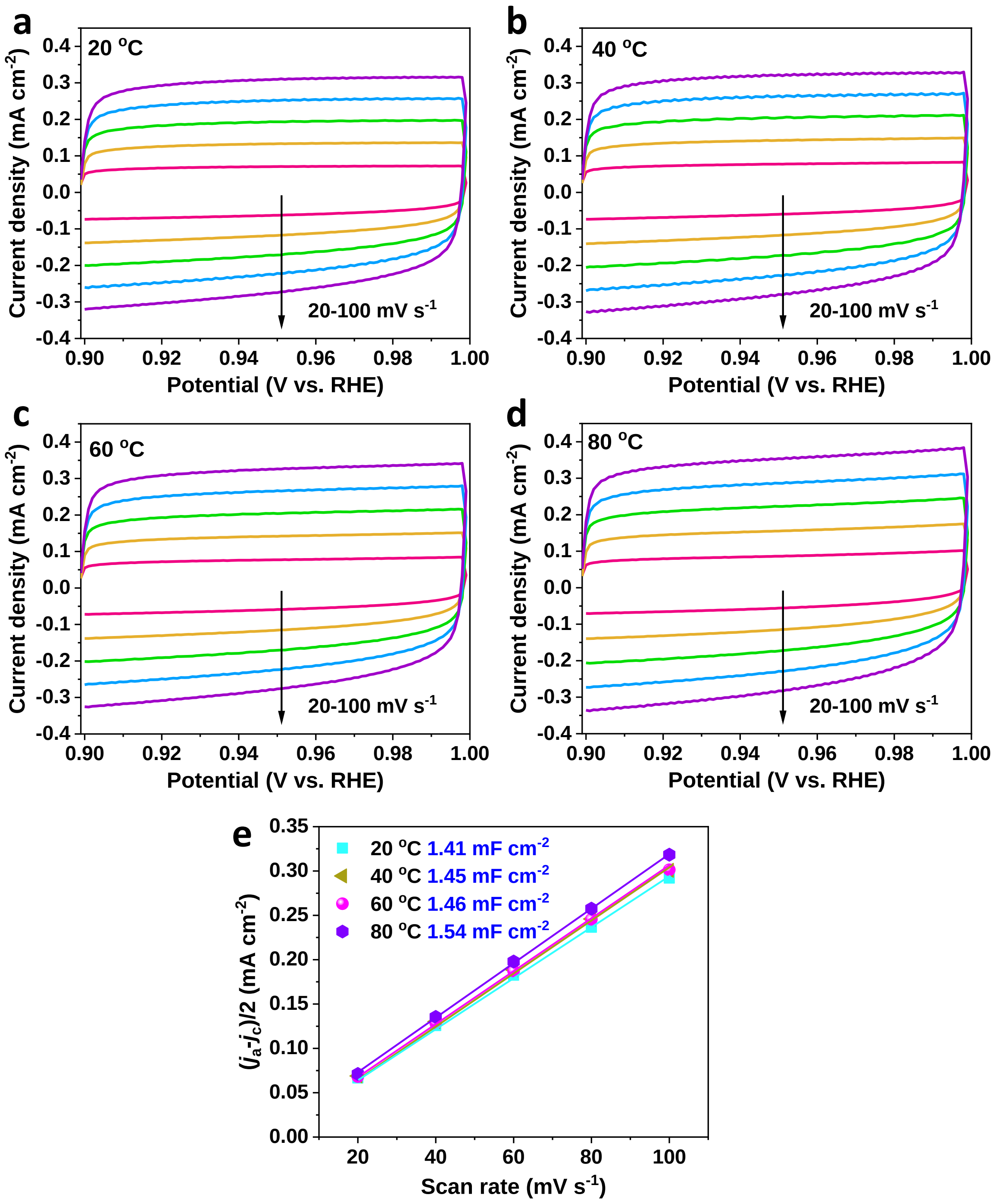

Supplementary Fig. 8 | a-d, CV curves of $SrIrO_3$ under different scan rates in non-faradic region under 20 °C **(a)**, 40 °C **(b)**, 60 °C **(c)**, and 80 °C **(d)**. **e,** The non-faraday current density at 0.95 V as a function of scan rates. The slope of fitting line is the double-layer capacitance.

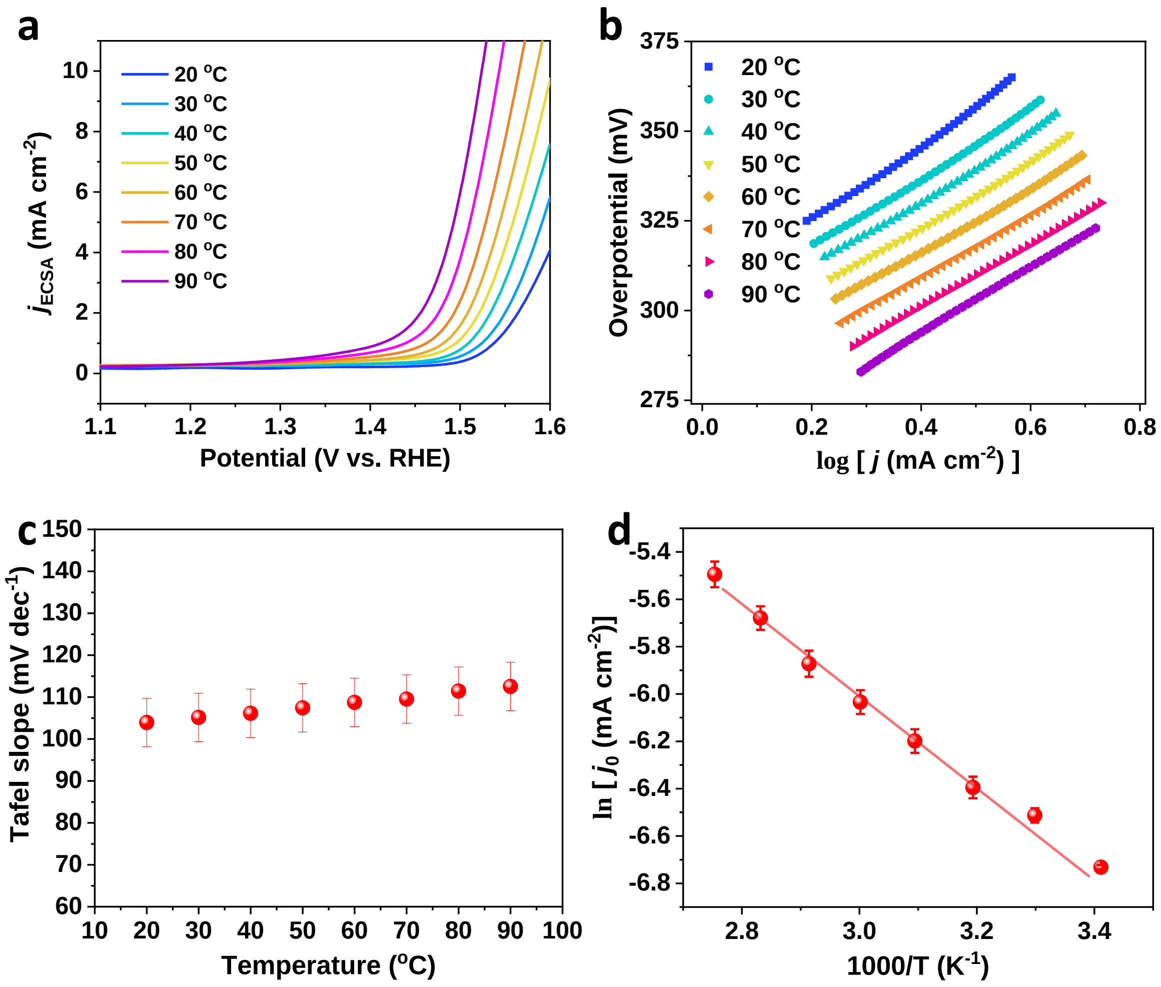

Supplementary Fig. 9 | a, LSV curves normalized by ECSA of $SrIrO_3$ in 1.0 M KOH under different temperatures. (scan rate, 5 mV/s; mass loading, 0.4 mg cm^{-2}; 90% *iR*-drop compensation is utilized; the pH of the electrolyte under different temperatures are shown in Supplementary Table 7.) **b,** Tafel slopes for OER on $SrIrO_3$ at different temperatures. **c,** Tafel slopes for OER on $SrIrO_3$ as a function of temperatures. **d,** The linear lnj_0 - 1/T Arrhenius relationship for OER on $SrIrO_3$.

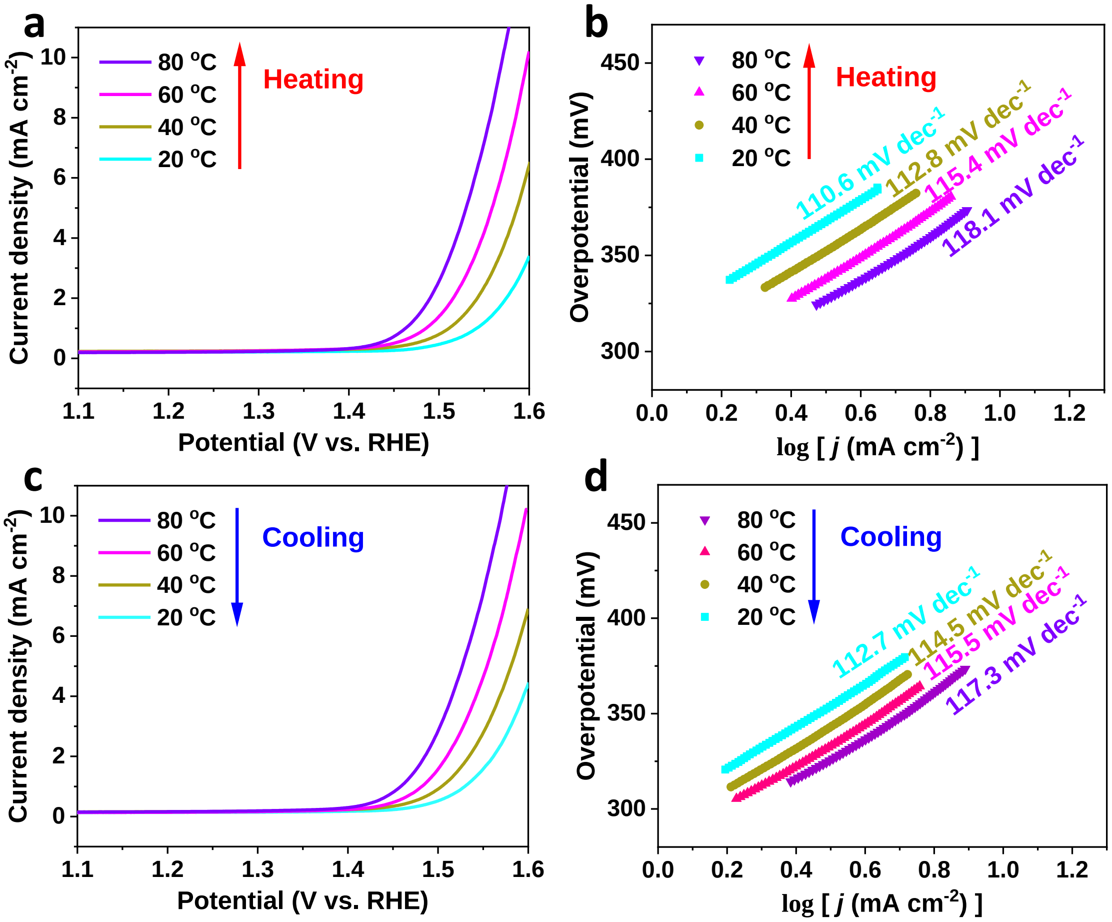

Supplementary Fig. 10 | a,b, LSV curves **(a)** and their Tafel slopes **(b)** for OER in 1.0 M KOH on $SrIrO_3$ during heating process from 20 °C to 80 °C. **c,d,** LSV curves **(c)** and their Tafel slopes **(d)** for OER in 1.0 M KOH on $SrIrO_3$ during cooling process from 80 °C to 20 °C. (scan rate, 5 mV/s; mass loading, 0.4 mg cm^{-2}; 90% *iR*-drop compensation is utilized; the pH of the electrolyte under different temperatures are shown in Supplementary Table 7.)

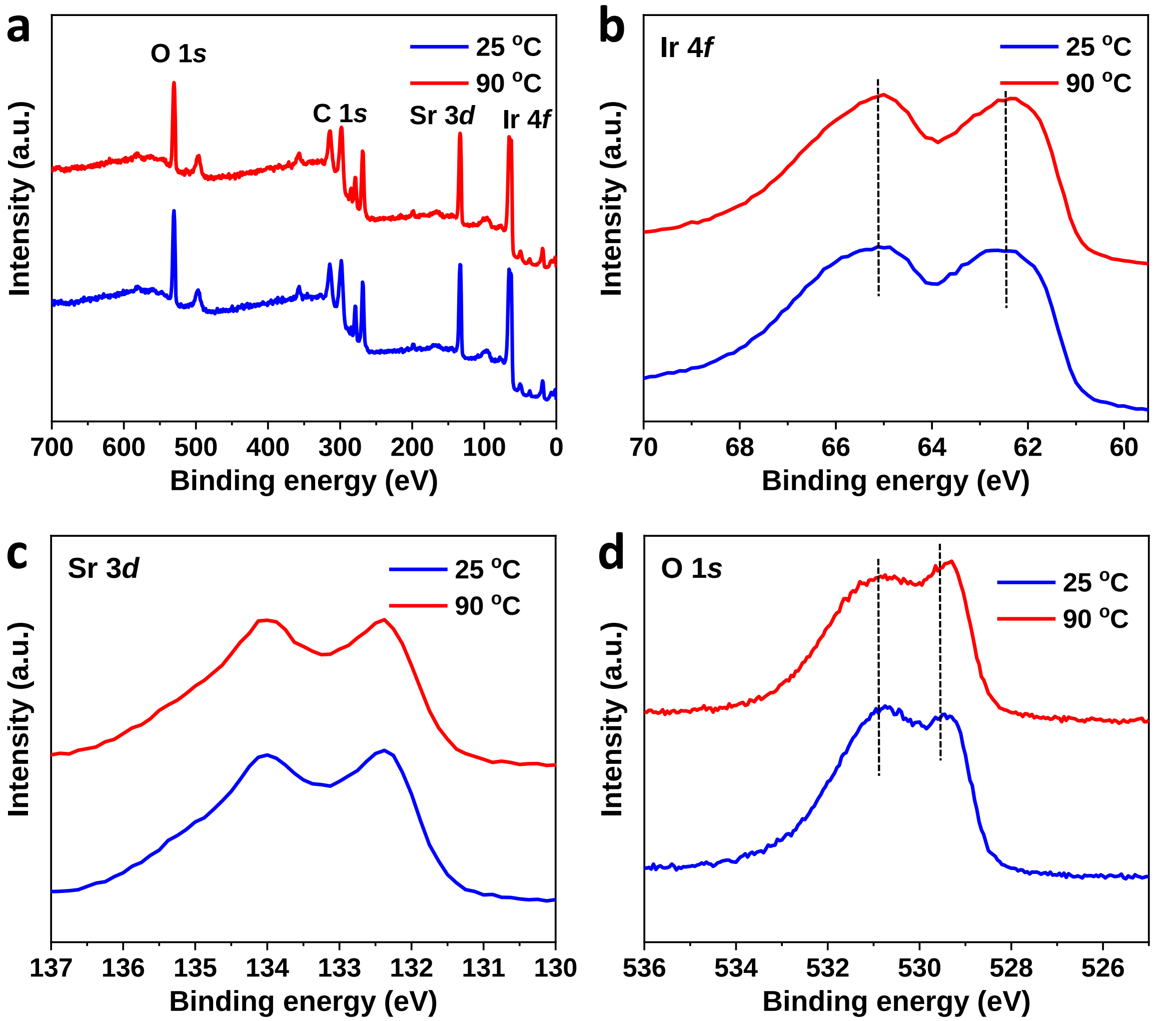

Supplementary Fig. 11 | a, Survey XPS spectra for the $SrIrO_3$ under 25 °C and 90 °C. **b-d,** The core-level XPS spectra of Ir 4*f* **(b)**, Sr 3*d* **(c)**, and O 1*s* **(d)**.

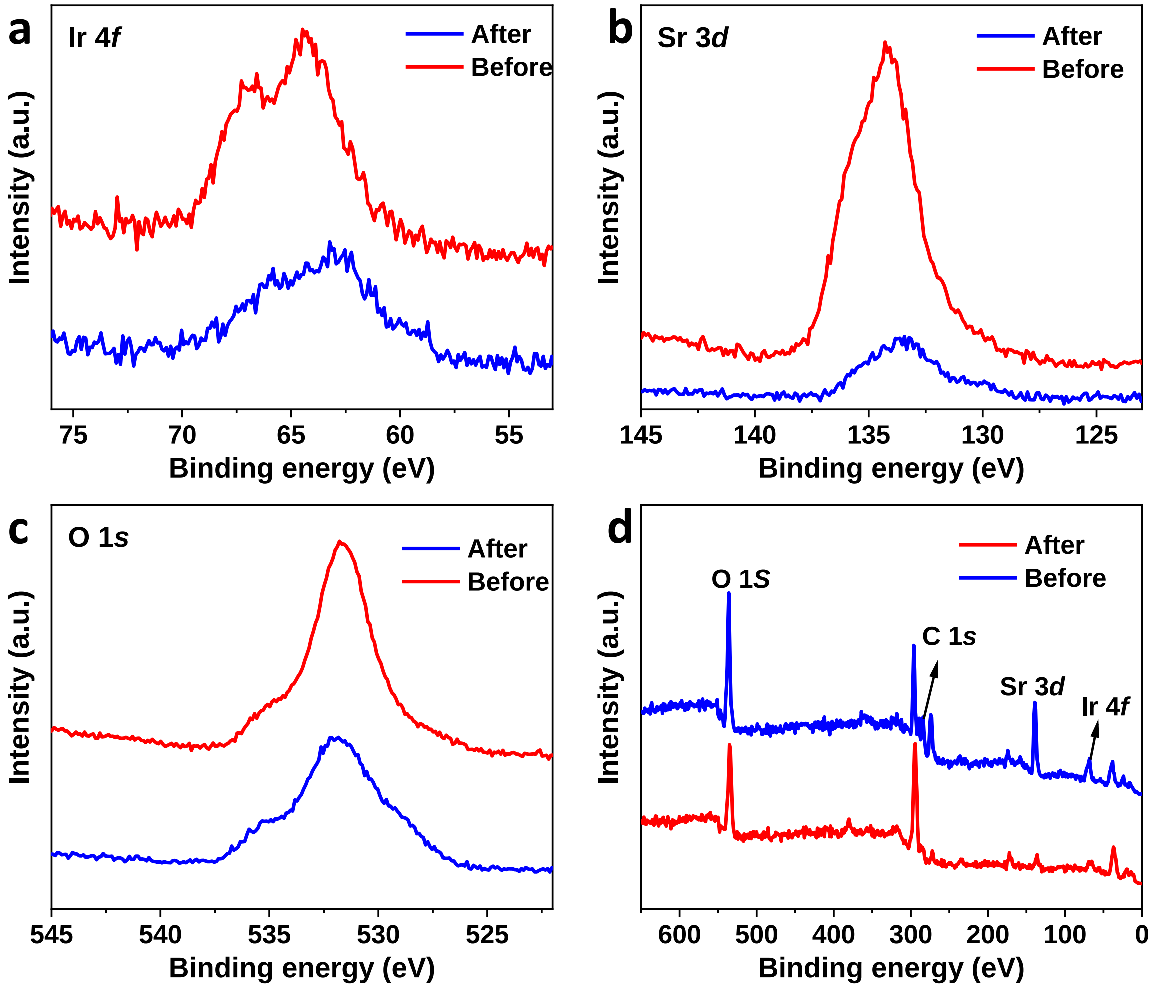

Supplementary Fig. 12 | XPS spectra for the Sr_2IrO_4 before and after OER under 90 ^oC. **a-c,** The core-level XPS spectra of Ir 4*f* **(a)**, Sr 3*d* **(b)**, and O 1*s* **(c)**. **d,** Survey XPS spectra for the Sr_2IrO_4 before and after OER under 90 ^oC.

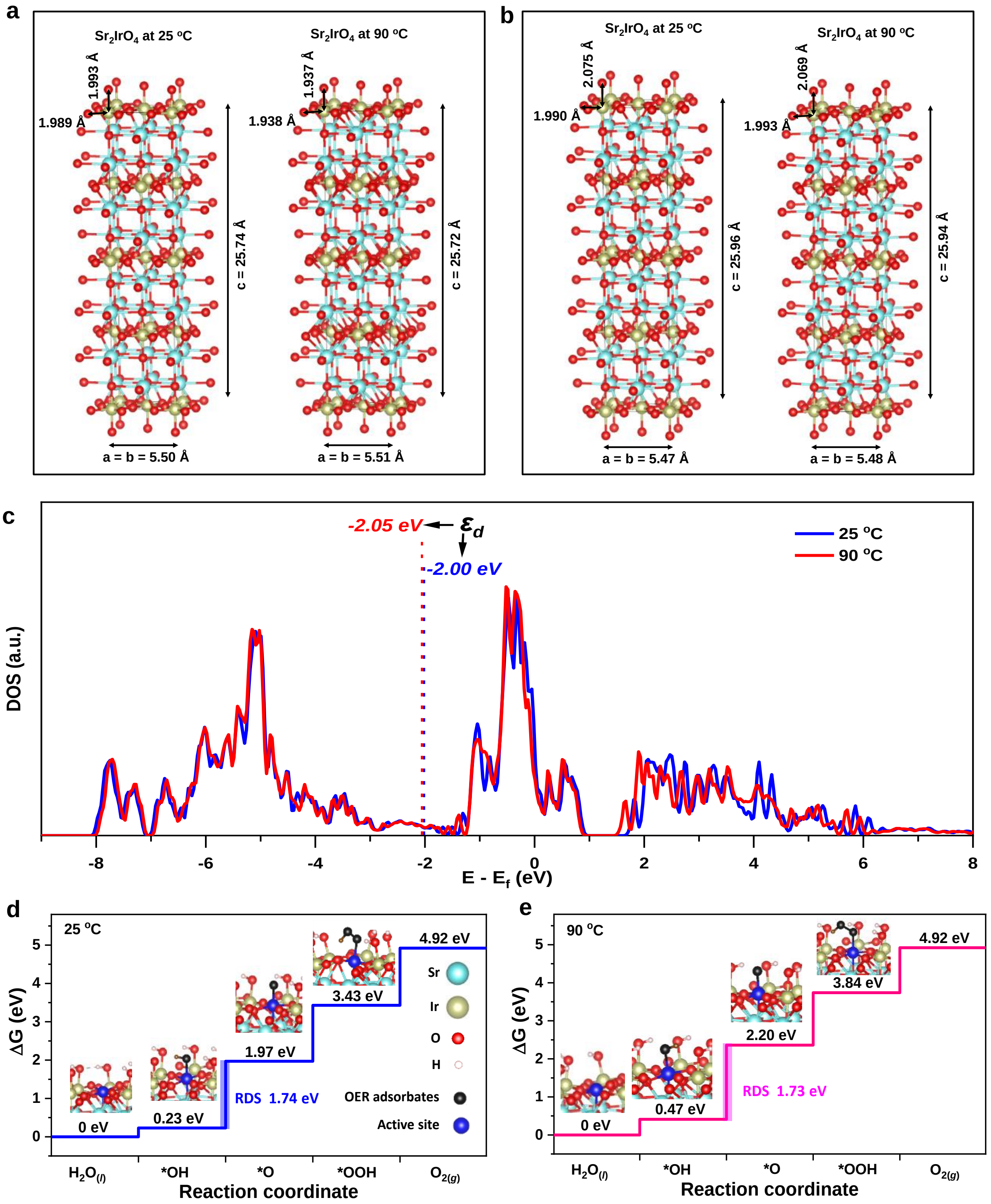

Supplementary Fig. 13 | Modeling the thermal strains for IrO_6 units in Sr_2IrO_4. a, To model the thermal strains in IrO_6 units, the crystal structure of Sr_2IrO_4 with $I4_1/acd$ symmetry was created by setting the cell parameters from XRD refinement and the atomic fractional coordinates from Ir-O bonding length in IrO_6 units from EXAFS analysis. **b,** The crystal structure of Sr_2IrO_4 was created according to the JCPDS No. 09-0099. Firstly, the structure is acquired through structure relaxing both of the lattice parameters and the atomic fractional

coordinates. Then, the thermal strains at 20 °C and 90 °C were considered by relaxing the atomic positions in the situation of fixed lattice parameters with a thermal expansion coefficient of 2.8×10^{-5} K^{-1} for *a*- and *b*-axes and -1.2×10^{-5} K^{-1} for *c*-axis, which is obtained by fitting XRD data. **c,** The projected DOS of Ir 5*d* for the Sr_2IrO_4 with a crystal structure created by setting thermal expansion coefficient to model the thermal strains under 25 °C and 90 °C (Structural model in Supplementary Fig. 13b). The ε_d indicates the *d*-band center calculated as the first statistical moment of the *d*-projected DOS. **d,e,** Gibbs free energy diagrams for adsorbed the OER intermediates ($^*OH$, $^*O$, $^*OOH$) onto Ir active site of (001) facet of Sr_2IrO_4 with a crystal structure created by setting thermal expansion coefficient to model the thermal strains under 25 °C (**d**) and 90 °C (**e**). The (001) slab model with six atomic layers in a 1×1 unit is created according to the Sr_2IrO_4 with a crystal structure created by setting thermal expansion coefficient to model the thermal strains under 25 °C and 90 °C. The vacuum regions of 15 Å were used to avoid periodic image interactions, that is, there is enough vacuum space so that the electron density of the material tails off to zero in the vacuum and the top of one slab has essentially no effect on the bottom of the next. We performed the hydroxyl group passivating the coordinately unsaturated Ir sites except the Ir active site on the slab model to stabilize the surface structure, thus avoiding the undesired surface reconstruction during structure optimization. The structure optimization was performed by fixing the bottom four layers and allowing the top two layers to relax. The single-site AEM model with 100% coverage of adsorbates on Ir active site was used to check the intrinsic catalytic activity (100% coverage is one adsorbate per one active site).[14]

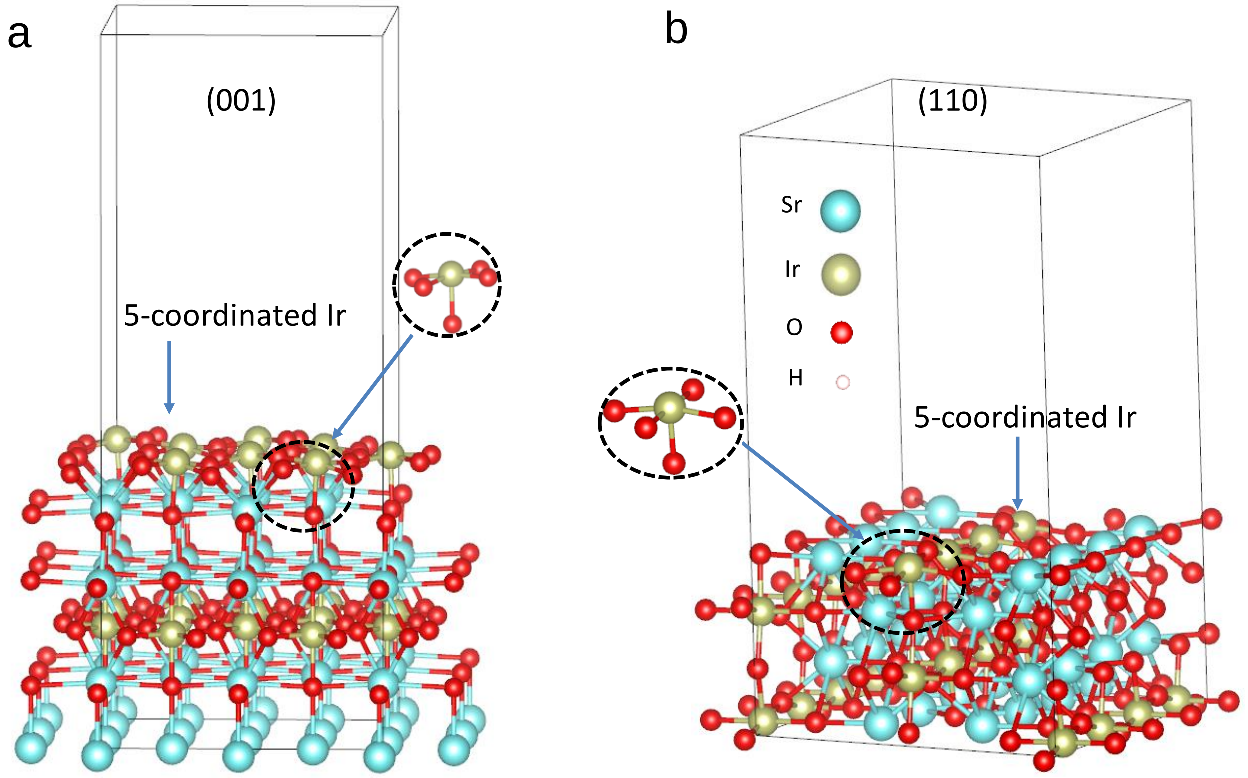

Supplementary Fig. 14 | The slab model for Sr_2IrO_4 (001) and (110) facets. a,b, The Ir coordination environment on the (001) (**a**) and (110) (**b**) facets.

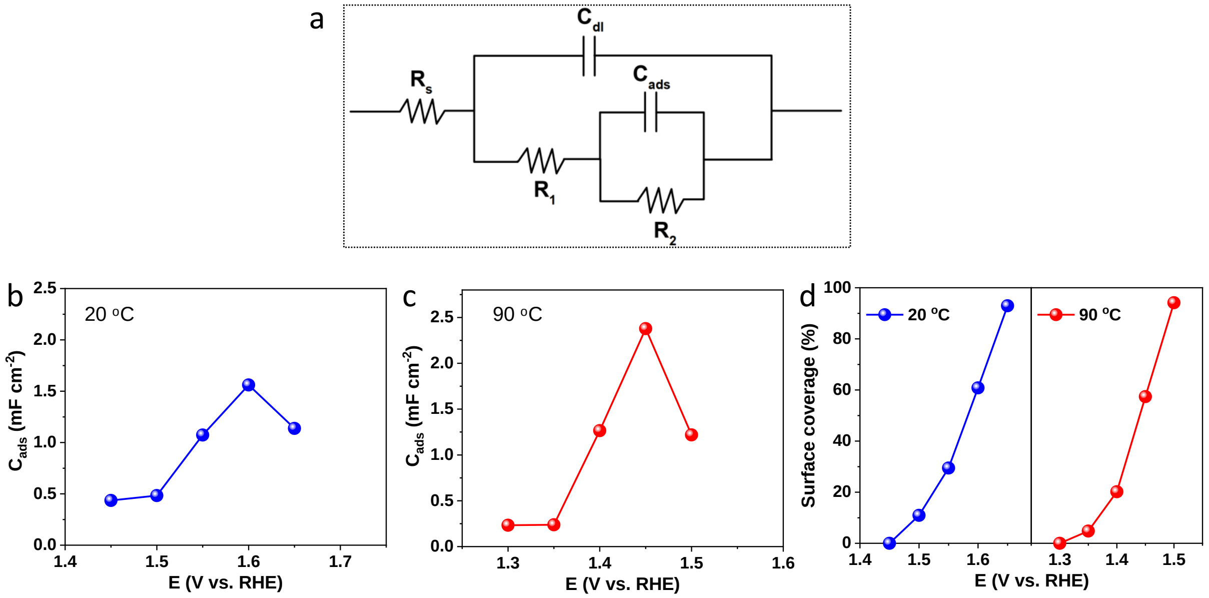

Supplementary Fig. 15 | Electrochemical characterizations of OER intermediates adsorption. a, The equivalent circuit used in EIS fitting. R_s refers to the series resistance in the circuit. C_{dl} and C_{ads} are the capacitance from double layer and intermediates adsorption, respectively, the charge-transfer resistance, R_{ct}, is composed of R_1 and R_2 ($R_{ct} = R_1 + R_2$).[1] **b,c** The C_{ads} from fitting EIS data (Fig. 3d,e) for Sr_2IrO_4 under 20 (**b**) and 90 °C (**c**), as a function of applied potentials. **d**, The calculated surface coverage for OER intermediates on Sr_2IrO_4 at around onset potential under 20 and 90 °C.

Surface coverage of OER intermediates at different temperatures was calculated by fitting the EIS plots recorded at different potentials. As shown in Supplementary Fig. 15a, the Randle's equivalent circuit is composed of the series resistance in the circuit (R_s), the capacitance from double layer (C_{dl}) and intermediates adsorption (C_{ads}), the charge-transfer resistance (R_1 and R_2, $R_{ct} = R_1 + R_2$).[2] In the EIS plot, the C_{ads} and C_{dl} were obtained by fitting the plots from low-frequency regions (Supplementary Fig. 15b,c).

Harrington and Conway gave the definition of potential-dependent $C_{ads}(E)$ as:[3]

$$C_{ads}(E) = \sigma\, [d\theta(E)/dE] \qquad (1)$$

where $C_{ads}(E)$ is the capacitance of intermediates adsorption at applied potential E, σ is the charge density for a monolayer coverage, which can be assumed as constant because the adsorption sites of Sr_2IrO_4 are stable during OER, Here, the σ was taken as 0.21 mC cm^{-2}, a typical value for a monolayer coverage on Pt plate electrode.[3] $\theta(E)$ is the surface coverage of OER intermediates at applied potential E.

By integrating Eq. 2, we can get the $\theta(E)$:

$$\theta(E) = [\int_{E_0}^{E} C_{ads}(E)\, dE]/\sigma \qquad (2)$$

where the E_0 refers to the potential under which no obvious adsorption occurs, the E is the potential at arbitrary coverage. In our case, the E_0 is 1.45 V vs. RHE under 20 ^{o}C and 1.3 V vs. RHE under 90 ^{o}C, respectively.

As shown in Supplementary Fig. 15d, the Sr_2IrO_4 exhibited the similar coverage of OER intermediates at the same current density, nearly independent of the temperatures, suggesting that the coverage of OER intermediates is independent of the temperatures.

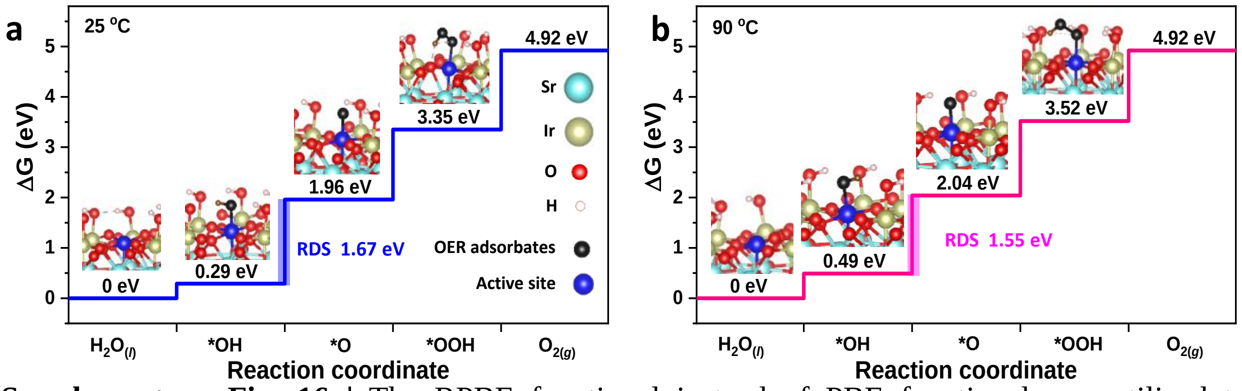

Supplementary Fig. 16 | The RPBE functional instead of PBE functional was utilized to calculate the chemisorption energies between Sr_2IrO_4 and OER intermediates. **a, b,** Gibbs free energy diagrams for OER intermediates (*OH, *O, *OOH) adsorbing onto the single Ir active site (the 100% coverage of OER intermediates was achieved by hydroxyl group passivating the coordinately unsaturated Ir sites except the single Ir active site) of the likely exposed Sr_2IrO_4 (001) surface with thermal strains under 25 °C **(a)** and 90 °C **(b)**, with calculated structures and rate-determining steps. Insets are the corresponding structures of the *OH, *O, and *OOH adsorptions on Ir sites. The Gibbs free energy changes were calculated without considering the effects of temperature, pH, and solvation on OER.

To more accurately calculate chemisorption energies, PBE functional was revised by Nørskov for improving the mathematical form for the exchange energy enhancement factor. However, the two functionals, PBE and RPBE, follow the same construction logic and therefore contain the same physics and fulfill the same physical criteria, and therefore the limitations of PBE still remain in RPBE.

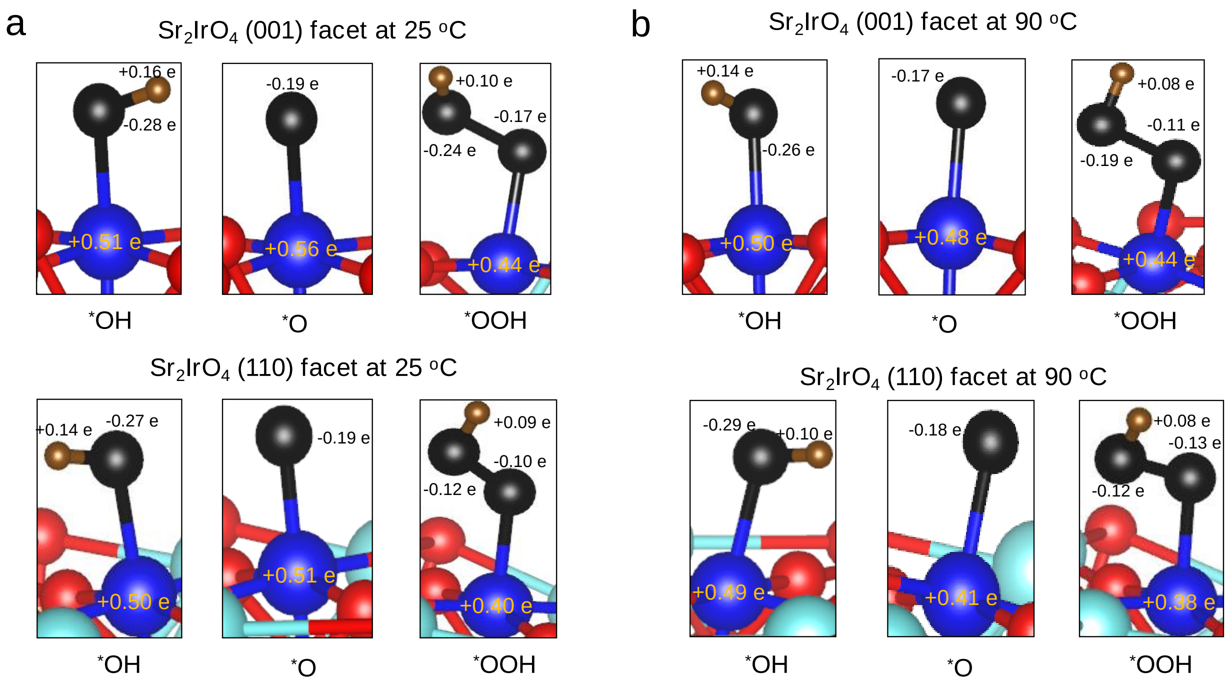

Supplementary Fig. 17 | The electron transfer number ($\Delta\rho$) was obtained by Bader charge analysis during OER intermediates (*OH, *O, *OOH) adsorbing onto Ir active site on different facets. **a,** (001) and (110) facets under 25 ^{o}C. **b,** (001) and (110) facets under 90 ^{o}C. The single-site adsorbate evolution model, achieving by OH^- passivating the coordinately unsaturated Ir sites on the terminal facet except the Ir active site, was used in the DFT calculations by setting the 100% coverage of all the OER intermediates adsorbing onto single Ir active site. The black ball is adsorbed oxygen atom. The blue ball is the Ir active site. The brown ball is the hydrogen atom.

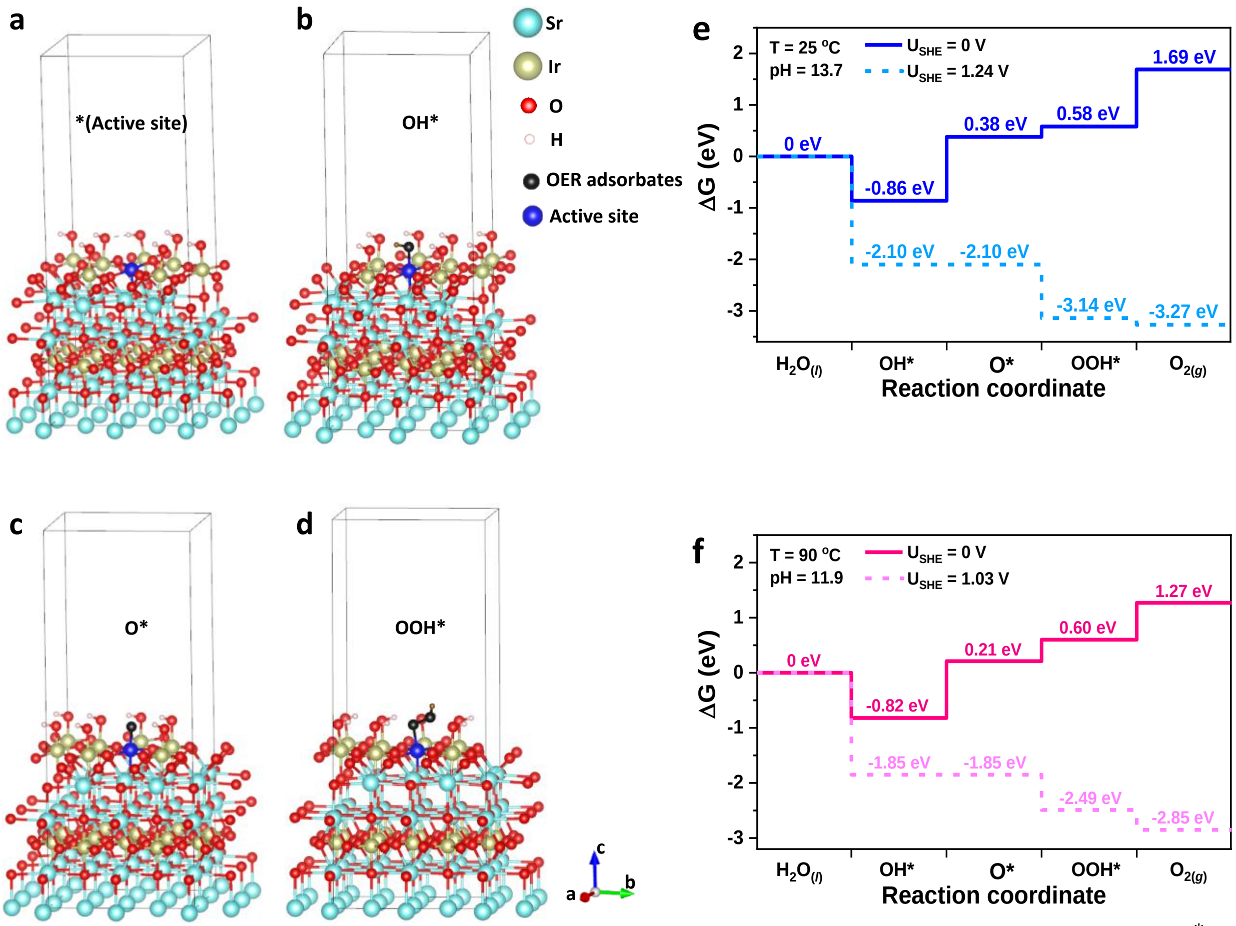

Supplementary Fig. 18 | a-d, Structures of the Ir sites of the Sr_2IrO_4 (001) surface **(a)** and $^*OH$ **(b)**, $^*O$ **(c)**, $^*OOH$ **(d)** adsorptions on Ir sites. **e,f,** Gibbs free energy diagrams for OER intermediates ($^*OH$, $^*O$, $^*OOH$) adsorbed on the Ir sites of the Sr_2IrO_4 (001) surface with thermal strains under 25 °C (**e**) and 90 °C (**f**). Solvation, temperature, and pH effects were included in the calculations. The single-site adsorbate evolution model, achieving by OH^- passivating the coordinately unsaturated Ir sites on the terminal facet except the Ir active site, was used in the DFT calculations by setting the 100% coverage of all the OER intermediates adsorbing onto single Ir active site. The implicit solvation model was used to describe the solvation effect due to spontaneous H_2O dissociation on Ir active site of Sr_2IrO_4 and the advantage of this model in describing the temperature effect, pH, and concentrations.

We calculated the standard Gibbs free energy for $^*OH$, $^*O$, and $^*OOH$ adsorbed on Sr_2IrO_4 by considering the active sites, pH, potential, temperature, and solvation effects. The unsaturated Ir sites on the surface were passivated by hydroxyl group.

Effects of temperature. As described in reference,[4] Rajan et al. have developed a method to describe standard Gibbs free energies of OER intermediates at arbitrary temperatures below the water boiling point. We adapted the same method to generalize the standard Gibbs free energies of molecular (H_2, O_2, and H_2O), ionic ($H^+ + e^-$) species, and the OER intermediates ($^*OH$, $^*O$, $^*OOH$) at any temperature T lower than the boiling point of water.

In our case, the OER thermodynamics at elevated temperature was calculated based on the computational hydrogen electrode (CHE) framework advanced by Nørskov and colleagues.[5]

Given that the CHE is based on the standard hydrogen electrode (SHE), whose potential at all temperatures is zero by convention, the standard potential of the hydrogen evolution reaction is zero in this framework, so that:

$$G^0_{(H^++e^-)}(T) = 0.5G^0_{H2(g)}(T) \quad (3)$$

where $G^0_{(H^++e^-)}(T)$ and $G^0_{H2(g)}(T)$ are the standard Gibbs free energies of the proton-electron couple and gaseous hydrogen at temperature T.

The standard Gibbs free energies of molecular H_2 and H_2O at arbitrary temperatures below the water boiling point were calculated by the Eq.4:

$$G_i^0(T) = E_i^{\mathrm{DFT}} + \mathrm{ZPE}_i^{\mathrm{DFT}} + [H_i^0(T) - H_i^0(0\ \mathrm{K}) - TS_i^0(T)] \quad (4)$$

where i denotes either H_2(g) or H_2O(g), E_i^{DFT} the species' DFT total energy at zero Kelvin, $\mathrm{ZPE}_i^{\mathrm{DFT}}$ its zero-point vibrational energy from DFT. $H_i^0(T)$ and $S_i^0(T)$ its standard enthalpy and entropy at T, respectively, and can be calculated through Shomate equations from the National Institute of Standards and Technology (NIST) database [https://dx.doi.org/10.18434/T4W30F].

The standard Gibbs free energy of liquid water at temperature T was calculated by

$$G^0_{H2O(l)}(T) = G^0_{H2O(g)}(T) + \Delta G^0_{c,H2O(g)}(T) \quad (5)$$

where $\Delta G^0_{c,H2O(g)}(T)$ is the standard Gibbs free energy of the condensation of water vapor. The standard Gibbs free energy of molecular O_2 was calculated by the following equation:

$$G^0_{O2(g)}(T) = \Delta G^0_{r,H2O\ split}(T) + [2G^0_{H2O(l)}(T) - 2G^0_{H2(g)}(T)] \quad (6)$$

where $\Delta G^0_{r,H2O\ split}(T)$ is the standard Gibbs free energy of the reaction for water splitting.

The free energies of adsorbed OER intermediates (*OH, *O, *OOH) as a function of temperature using the harmonic oscillator model for solids as:

$$G_i^0(T) = E_i^{\mathrm{DFT}} + E_{\mathrm{vib},i} - TS_{\mathrm{vib},\,i} \quad (7)$$

where G_i^0 is the standard Gibbs free energy of the ith OER intermediates, E_i^{DFT} is the total energy of the ith OER intermediates, $E_{\mathrm{vib},i}$ is vibrational internal energy, $TS_{\mathrm{vib},\,i}$ is vibrational entropy.

Effects of pH. In our case, the pH changes of the electrolyte are originated from the thermally adjusted ability of water to ionise. When heating, pH of the electrolyte decreases from 13.7 at room temperature to 11.9 at 90 ^{o}C with no change in KOH concentration to be 1.0 M. Therefore, in our case, the pH decrease mainly originates from the water dissociation of $H_2O = H^+ + OH^-$ driven by absorbing extra heat. The water ionisation constant increases with increasing the temperatures, thus decreasing the pH due to that the pH is defined as the negative log of the hydrogen ion concentration expressed in mol/L. We add the pH $\times k_BT\ln10$ into the Gibbs free energy expression of elementary reaction steps to reflect the effects of pH.

Solvation effects. When we treat the solvent effects, the two fundamentally different approaches to modeling this aqueous solution environment are implicit and explicit solvation models.[6] Implicit solvation methods approximate the solvent through a continuum model based

on electrostatics. Although this approach works well for long-range effects, the method cannot account for the immediate vicinity of an adsorbate in contact with a protic solvent such as liquid water, which interacts significantly with solutes through hydrogen bonds and van der Waals interactions. Explicit solvation methods can employ one or several layers of water molecules in a DFT calculation to compute the binding energies of adsorbates. These water layers are placed above the surface and can be ice-like or undergo structural relaxation. If the water layer is allowed to relax, a doubt remains whether the optimized water structure is an accurate representation of the liquid at the solvated interface. In any case, DFT structural relaxations are performed at 0 K, and therefore such schemes fail in describing the finite temperature behavior of liquid solvents. Furthermore, relaxation of the water layer near the adsorbate can lead to an overestimate of the stabilization due to hydrogen bonds, some of which can be disrupted in finite temperature dynamics where disorder is taken into account. This means that the explicit solvation model with relatively high uncertainty is more complex than the implicit solvation model, in particular, to consider the various factors including the concentrations, pH, potential, and temperature may increase the uncertainty of this approach.

In addition, usually, for alkaline water electrolysis on surface of oxides, the water dissociation is a spontaneous process to form ^{-}OH on the unsaturated sites. As demonstrated on the surface of IrO_2.[7] For the stoichiometric IrO_2 (110) surface, half of the surface Ir atoms are 5 coordinated. The H_2O binds strongly (by ~1.7 eV/H_2O in liquid water) at this surface and the H_2O molecules spontaneously dissociate to form ^{-}OH at the unsaturated Ir (Ir-5c) and ^{-}OH at the bridging O, independent of the starting surface water configurations. Indeed, in the alkaline water electrolysis, oxides or hydroxides have been demonstrated to be the efficient catalysts to accelerate water dissociation. For example, the alkaline water electrolysis on Pt electrode is effectively promoted by the Fe, Co, Ni, Mn containing hydr(oxy)oxides.[8,9] This means that the initial water dissociation on surface of oxides is commonly spontaneous process to occur, thus the implicit solvation model is able to describe the alkaline solvation environment for checking the evolution of OER intermediates ($^{*}OH$, $^{*}O$, $^{*}OOH$).

Accordingly, after we give comprehensive consideration to the pros and cons of explicit and implicit solvation model and the spontaneous H_2O dissociation on the surface of oxides, the implicit solvation model, approximating the solvent through a continuum model based on electrostatics, was adopted to consider the solvation effect involved with the effects of temperature, pH, and concentrations. In the implicit solvation model, the effects of temperature, pH, and concentrations on solvation effects can be considered by setting the relative permittivity of water and the Debye length of the 1.0 M electrolyte at different temperatures.

We utilize the implicit solvation model to describe the solvation effects under thermal stimulation through solving the linearized Poisson-Boltzmann equation by $VASP_{sol}$.[10,11] The relative permittivity of water was set as 78.4 under 25 ^{o}C and 58.13 under 90 ^{o}C.[12] The Debye length of the 1.0 M electrolyte was set to 3.04 Å under 25 ^{o}C and 3.36 Å under 90 ^{o}C.[10,11]
Considering the pH, potential, temperature, and solvation effects, the standard Gibbs free energy for elementary reaction steps at arbitrary temperatures below the water boiling point were calculated by Eqs. 8-10:

$$\Delta G_{*OH}(T) = G_{*OH}(T) + 0.5 \times G_{H2}(T) - G_{H2O}(T) - G_{*}(T) - eU + \text{pH} \times k_B T \ln 10 \quad (8)$$

$$\Delta G_{*O}(T) = G_{*O}(T) + G_{H2}(T) - G_{H2O}(T) - G_{*}(T) - eU + \text{pH} \times k_B T \ln 10 \quad (9)$$

$$\Delta G_{*OOH}(T) = G_{*OOH}(T) + 1.5 \times G_{H2}(T) - 2 \times G_{H2O}(T) - G_{*}(T) - eU + \text{pH} \times k_B T\ln 10 \qquad (10)$$

where, $-eU$ stands for the free energy changes for one electron transfer, U is electrode potential respect to the standard hydrogen electrode. pH effects on free energy can be defined as pH $\times$ $k_B T$ln10, where k_B is Boltzmann constant.[13]

Considering the pH, potential, temperature, and solvation effects (Supplementary Fig. 18), the Gibbs free energy profile on the Ir sites of the Sr_2IrO_4 (001) surface still follows the same trend as the energy calculations with sole consideration of the strain effect (**Fig. 4a,b)**. The rate-determining step is still *OH deprotonation step with a smaller free energy difference between ΔG_{*OH} and ΔG_{*O} to be 1.24 eV under 25 ^{o}C and 1.03 eV under 90 ^oC.

Active sites on Sr_2IrO_4 with consideration of operation conditions. We calculated the Gibbs free energy diagrams for OER intermediates (*OH, *O, *OOH) adsorbed on the Sr or Ir sites on the Sr_2IrO_4 surface. The results were shown in Supplementary Figs. 18 and 19 and indicated that the Ir sites are more likely to be the active OER catalytic center due to the lower energy requirement in rate-determining step. The RDS on Sr sites are estimated to be 3.62 and 3.38 eV under 25 and 90 ^oC, respectively, which both are much larger than on Ir sites.

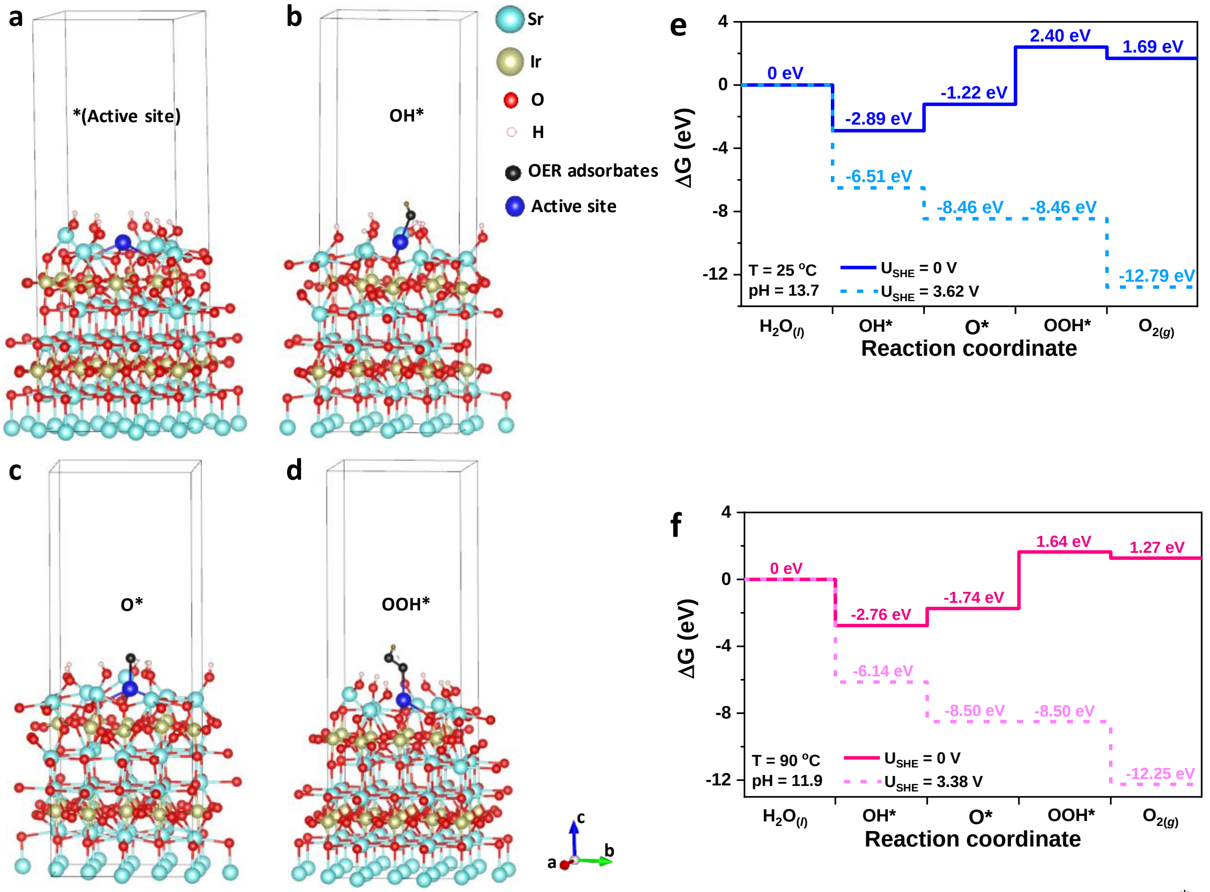

Supplementary Fig. 19 | a-d, Structures of the Sr sites of the Sr_2IrO_4 (001) surface **(a)** and $^*OH$ **(b)**, $^*O$ **(c)**, $^*OOH$ **(d)** adsorptions on Sr sites. **e,f,** Gibbs free energy diagrams for OER intermediates ($^*OH$, $^*O$, $^*OOH$) adsorbed on the Sr sites of the Sr_2IrO_4 (001) surface with thermal strains under 25 ^oC **(e)** and 90 ^oC **(f)**. Solvation, temperature, and pH effects were included in the calculations. The single-site adsorbate evolution model, achieving by OH^- passivating the coordinately unsaturated Sr sites on the terminal facet except the Sr active site, was used in the DFT calculations by setting the 100% coverage of all the OER intermediates adsorbing onto single Sr active site. The implicit solvation model was used to describe the solvation effect due to spontaneous H_2O dissociation on Sr active site of Sr_2IrO_4 and the advantage of this model in describing the temperature effect, pH, and concentrations.

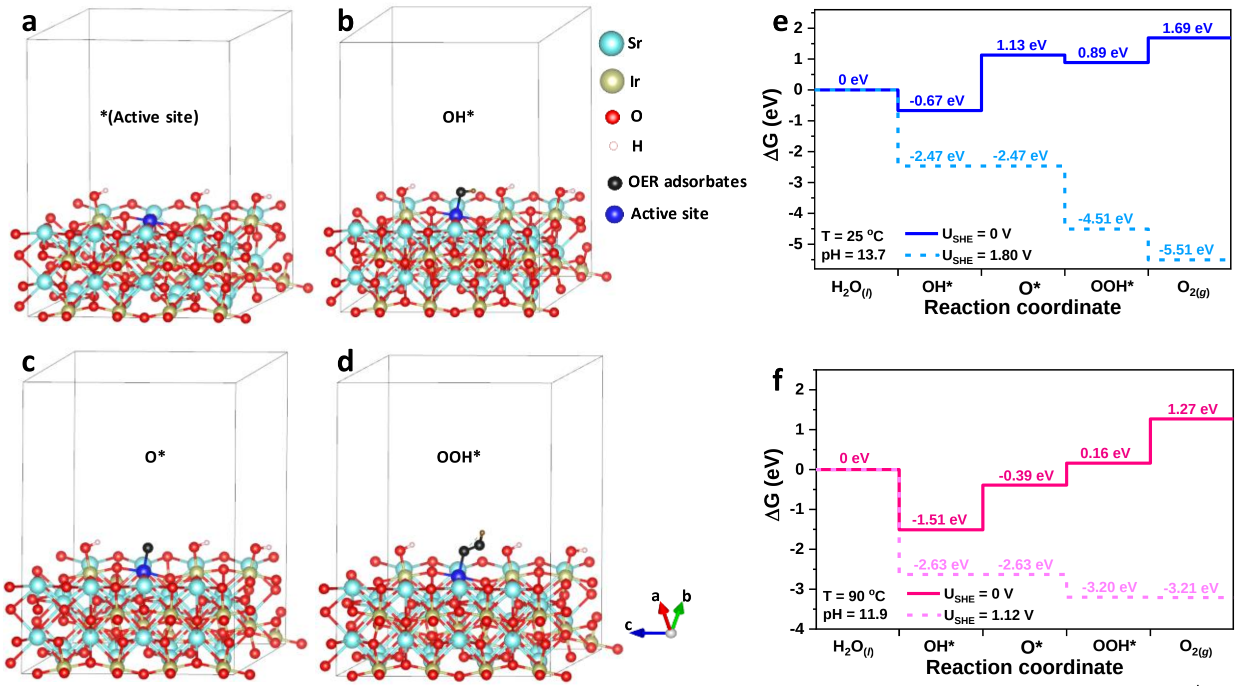

Supplementary Fig. 20 | a-d, Structures of the Ir sites of the Sr_2IrO_4 (110) surface **(a)** and $^*OH$ **(b)**, $^*O$ **(c)**, $^*OOH$ **(d)** adsorptions on Ir sites. **e, f,** Gibbs free energy diagrams for OER intermediates ($^*OH$, $^*O$, $^*OOH$) adsorbed on the Ir sites of the Sr_2IrO_4 (110) surface with thermal strains under 25 ^oC **(e)** and 90 ^oC **(f)**. Solvation, temperature, and pH effects were included in the calculations. The single-site adsorbate evolution model, achieving by OH^- passivating the coordinately unsaturated Ir sites on the terminal facet except the Ir active site, was used in the DFT calculations by setting the 100% coverage of all the OER intermediates adsorbing onto single Ir active site. The implicit solvation model was used to describe the solvation effect due to spontaneous H_2O dissociation on Ir active site of Sr_2IrO_4 and the advantage of this model in describing the temperature effect, pH, and concentrations.

To determine the free energy of intermediates at the (110) surface of the Sr_2IrO_4 on the Ir site during the OER process, vacuum regions of 15 Å were used for (110) surface model, a 1 × 1 supercell. We calculated the OER activity on a single active site model, where the coverage for an intermediate adsorbate on the active site is 100%. To obtain a stable structure of (110) facet with the Ir terminated, the unsaturated Ir sites except the Ir active site were passivated by hydroxyls.

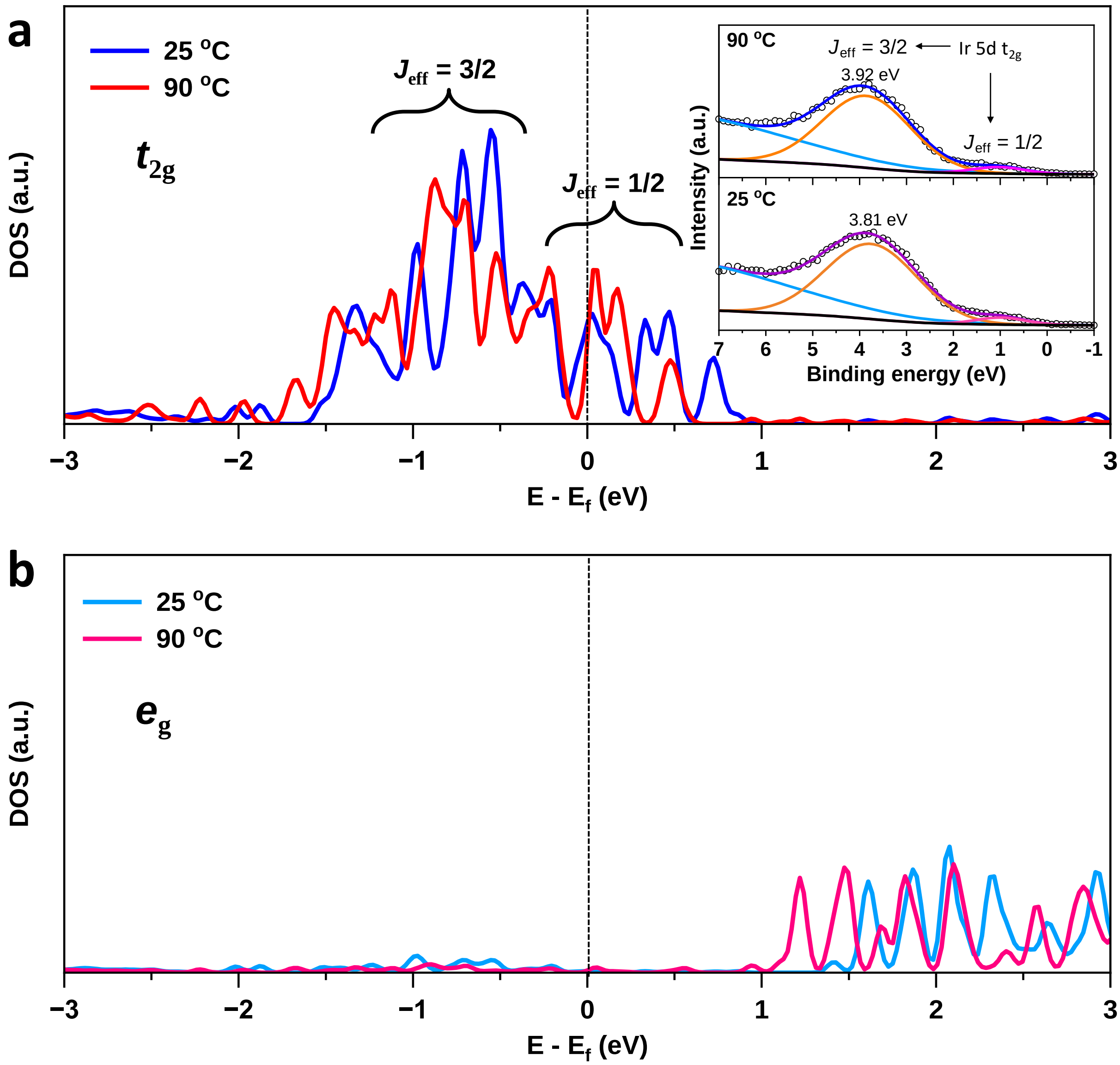

Supplementary Fig. 21 | a,b, Computed partial DOS of Ir 5*d* t_{2g} **(a)** and e_g **(b)** of Sr_2IrO_4 under different temperatures. Inset in Supplementary Fig. 19a shows the valence band spectra of Sr_2IrO_4 under different temperatures. The computed DOS of *d* band of Sr_2IrO_4 is in good accord with the XPS valence band analysis, suggesting that the GGA-PBE+*U*+SOC is acceptable for theoretical calculations of Sr_2IrO_4.

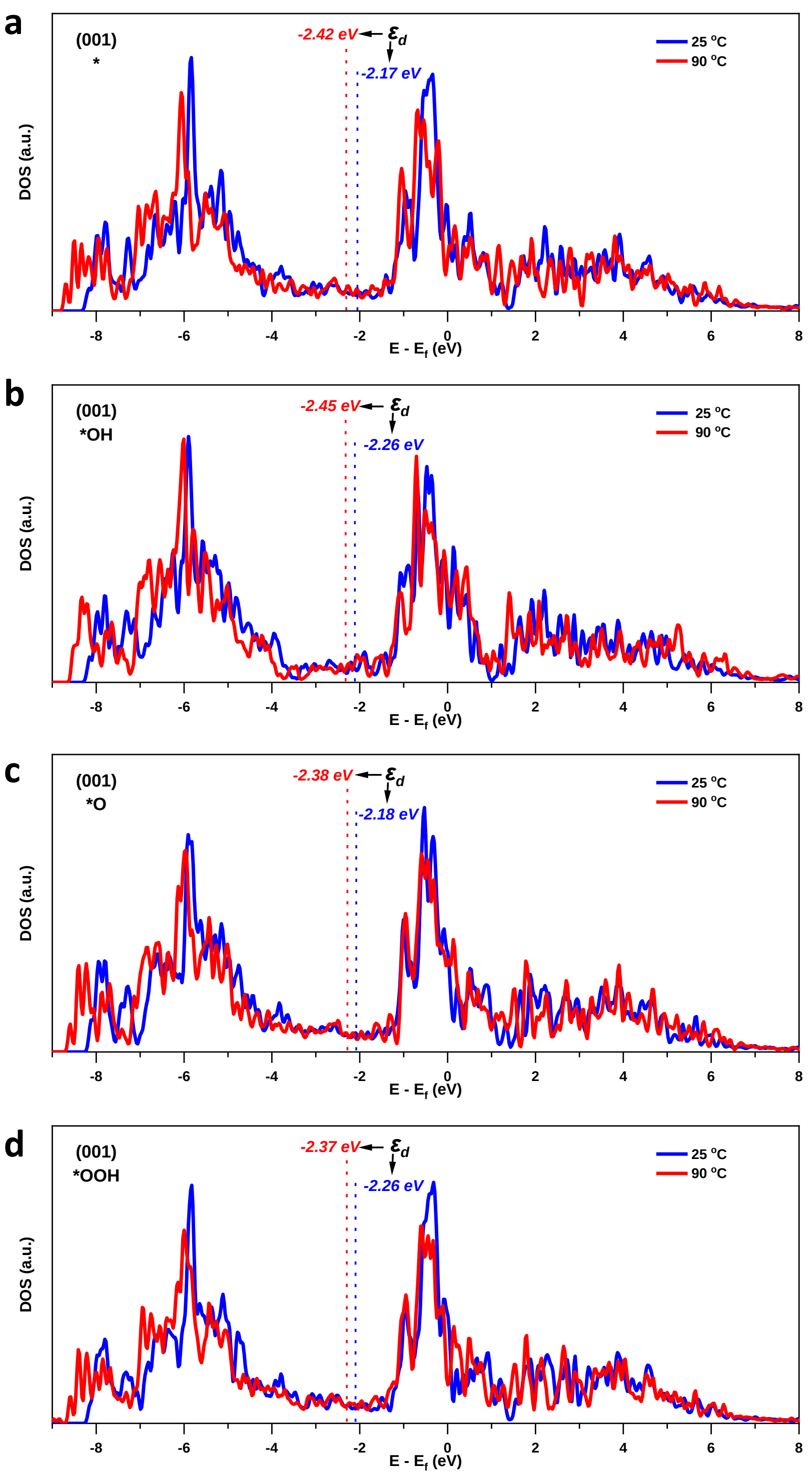

Supplementary Fig. 22 | The projected DOS of Ir 5*d* for Sr_2IrO_4 (001) facet after adsorption of *OH, *O, or *OOH onto Ir active site. **a,** Ir active site without adsorbates. **b,** *OH adsorption. **c,** *O adsorption. **d,** *OOH adsorption. The single-site adsorbate evolution model was used in the DFT calculations by setting the 100% coverage of all the OER intermediates adsorbing onto single Ir active site.

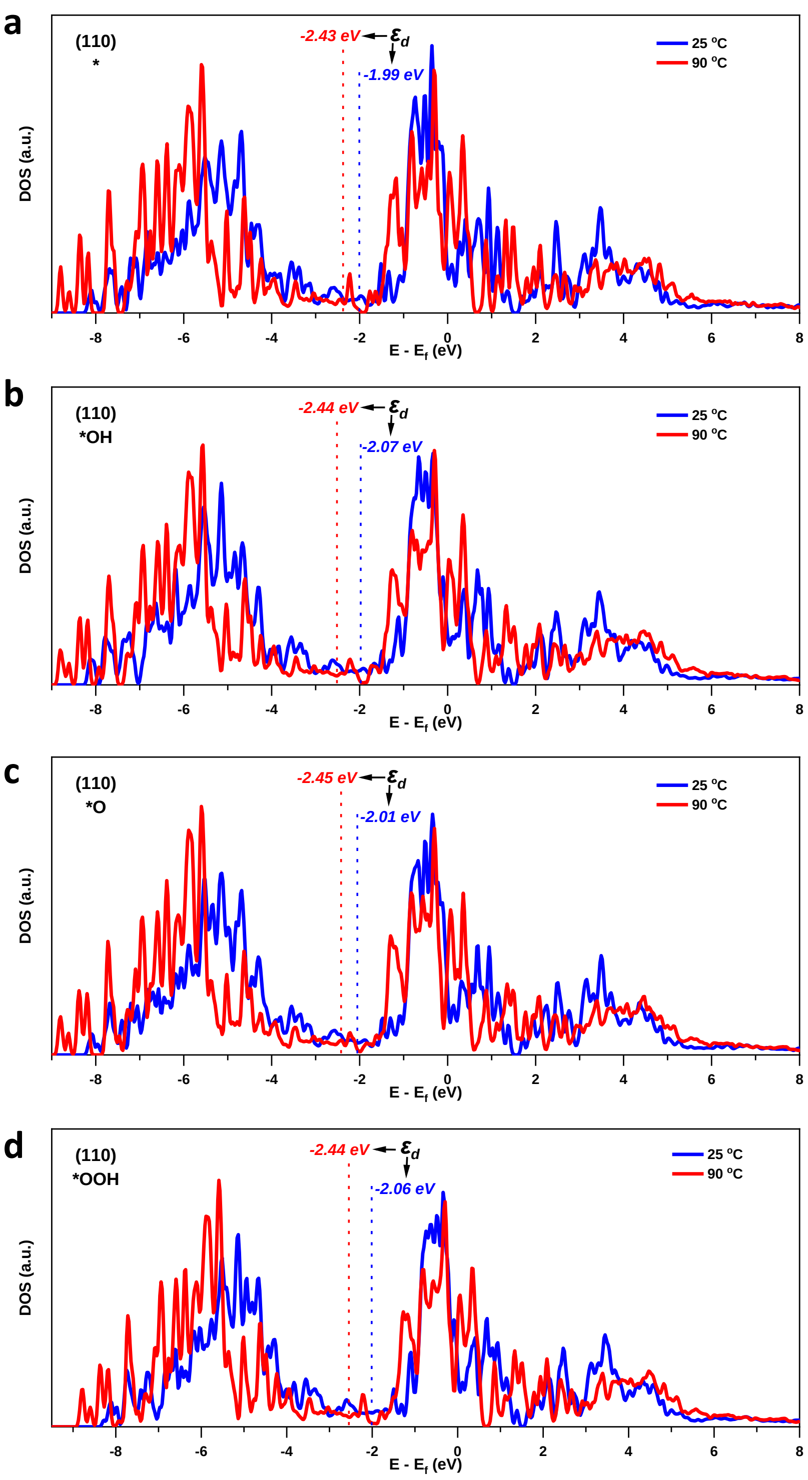

Supplementary Fig. 23 | The projected DOS of Ir 5*d* for Sr_2IrO_4 (110) facet after adsorption of *OH, *O, or *OOH onto Ir active site. **a,** Ir active site without adsorbates. **b,** *OH adsorption. **c,** *O adsorption. **d,** *OOH adsorption. The single-site adsorbate evolution model was used in the DFT calculations by setting the 100% coverage of all the OER intermediates adsorbing onto single Ir active site.

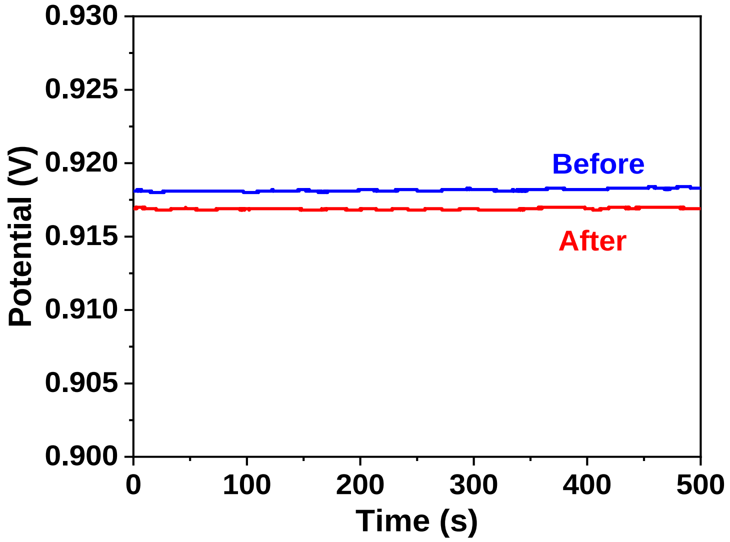

Supplementary Fig. 24 | The open-circuit voltage testing of the reference electrode before and after operating under different temperatures with the standard Hg/HgO as reference electrode at room temperature.

Supplementary Table 1. Fitting parameters for Raman spectra recorded under different temperatures.

Temperature/^{o}C	Mode	Position/cm^{-1}	Area	FWHM/cm^{-1}
25	v_1	168.20	5936.72	33.68
	v_2	307.31	3244.93	41.99
	v_3	383.59	927.16	27.47
	v_4	525.05	9034.50	48.64
60	v_1	167.27	9455.92	33.88
	v_2	311.85	4170.43	36.26
	v_3	382.65	1883.96	31.64
	v_4	526.16	14644.96	50.96
90	v_1	167.15	9921.34	35.49
	v_2	314.12	4176.54	39.96
	v_3	380.05	1120.43	23.99
	v_4	525.17	17775.87	55.44

Supplementary Table 2. EXAFS fitting parameters at Ir L_3-edge for Sr_2IrO_4 under 25 and 90 ^{o}C (S_0^2=0.98).

Temperature/^{o}C	Shell	*CN* [a]	**R**/Å [b]	σ^2/Å^2 [c]	ΔE_0 [d]	*R* factor
25	Ir-$O_{c\text{-}axis}$	2	1.993	0.0007	9.90	0.0103
	Ir-$O_{ab\text{-}plane}$	4	1.989	0.0007	9.90	
	Ir-Ir	4	2.730	0.0013	9.90	
	Ir-Sr	8	2.761	0.0116	9.90	
90	Ir-$O_{c\text{-}axis}$	2	1.969	0.0007	9.93	0.0099
	Ir-$O_{ab\text{-}plane}$	4	1.965	0.0007	9.93	
	Ir-Ir	4	2.744	0.0007	9.93	
	Ir-Sr	8	2.772	0.0010	9.93	

[a]*CN*, coordination number; [b]**R**, distance between absorber and backscatter atoms; [c]σ^2, Debye-Waller factor to account for both thermal and structural disorders; [d]ΔE_0, inner potential correction; *R* factor indicates the goodness of the fit.

Supplementary Table 3. The fitting parameters of the Ir 4*f* XPS spectra.

Temperature	Element / Transition	Peak Energy / eV	Peak Width FWHM / eV	Peak Area / eV counts
25 ^{o}C	Ir 4$f_{5/2}$	61.81	2.33	71308
	Ir 4$f_{5/2}$ satellite	63.15	2.78	40123
	Ir 4$f_{7/2}$	64.61	2.33	53481
	Ir 4$f_{7/2}$ satellite	65.95	2.78	30092
90 ^{o}C	Ir 4$f_{5/2}$	61.98	2.26	66216
	Ir 4$f_{5/2}$ satellite	63.41	2.63	30909
	Ir 4$f_{7/2}$	64.78	2.26	49662
	Ir 4$f_{7/2}$ satellite	66.21	2.63	23182

Supplementary Table 4. The fitting parameters of the valence band spectra.

Temperature	Element / Transition	Peak Energy / eV	Peak Width FWHM / eV	Peak Area / eV counts
25 ^{o}C	Ir 5*d* t_{2g} J_{eff} = 1/2	0.99	1.23	428.95
	Ir 5*d* t_{2g} J_{eff} = 3/2	3.76	2.30	8581.4
	Ir-O π band	8.17	5.86	14237
90 ^{o}C	Ir 5*d* t_{2g} J_{eff} = 1/2	1.03	1.29	383.53
	Ir 5*d* t_{2g} J_{eff} = 3/2	3.85	2.21	7208.9
	Ir-O π band	8.17	5.74	11552

Supplementary Table 5. The temperature-dependent theoretical thermodynamic water splitting potential (E^{o}_{H2O}).[15]

Temperature / ^{o}C	E^{o}_{H2O} / V vs. RHE
20	1.2300
30	1.2245
40	1.2155
50	1.2065
60	1.1975
70	1.1885
80	1.1795
90	1.1715

Supplementary Table 6. Comparison of OER catalytic performance of Ir-based materials in recent reports

**Catalyst**	**Current density / mA cm^{-2}**	**Overpotential / mV**	**Electrolyte**	**Operation temperature**	**Ref.**
Sr_2IrO_4	10	312	1 M KOH	Room temperature	This work
Sr_2IrO_4	10	235	1 M KOH	90 ^{o}C	This work
$SrIrO_3$	0.1	270	0.1 M KOH	Room temperature	16
Sr_4IrO_6	10	287	0.1 M $HClO_4$	Room temperature	17
Sr_2IrO_4	10	286	0.1 M $HClO_4$	Room temperature	17
$SrIrO_3$	10	353	0.1 M $HClO_4$	Room temperature	17
Sr_2IrO_4	10	300	0.1 M $HClO_4$	Room temperature	18
$SrIrO_3$	10	280	0.5 M H_2SO_4	Room temperature	19
$SrIrO_3$	10	290	1 M KOH	Room temperature	19
$SrIrO_3$	10	300	1 M KOH	Room temperature	20
IrO_2	10	295	0.1 M $HClO_4$	Room temperature	21

Supplementary Table 7. The effects of temperature on reference electrode potential and pH in 1.0 M KOH.

Temperature / ^{o}C	Hg/HgO vs. RHE / V [a]	pH [b]
20	0.9170	13.9
30	0.9158	13.7
40	0.9126	13.4
50	0.9078	13.1
60	0.9040	12.8
70	0.8988	12.5
80	0.8929	12.2
90	0.8850	11.9

[a] The potential experimentally corrected by detecting the open circuit voltage in two electrode system comprising of a Hg/HgO electrode and a standard hydrogen electrode at the given temperatures.

[b] The pH of the electrolyte at a given temperature was measured by a pH meter (PHS - 3E, Shanghai INESA Scientific Instrument Co., Ltd) with temperature corrected.

Supplementary References

1 Lyons, M. E. G. & Brandon, M. P. The significance of electrochemical impedance spectra recorded during active oxygen evolution for oxide covered Ni, Co and Fe electrodes in alkaline solution. *J. Electroanal. Chem.* **631**, 62-70 (2009).

2 Tao, H. B., et al. Identification of surface reactivity descriptor for transition metal oxides in oxygen evolution reaction. *J. Am. Chem. Soc.* **138**, 9978-9985 (2016).

3 Harrington, D. A. & Conway, B. E. Kinetic theory of the open-circuit potential decay method for evaluation of behavior of adsorbed intermediates: Analysis for the case of the H_2 evolution reaction. J. Electroanal. Chem. Interfacial Electrochem. **221**, 1-21 (1987).

4 Govind Rajan, A., Martirez, J. M. P. & Carter, E. A. Coupled effects of temperature, pressure, and ph on water oxidation thermodynamics and kinetics. *ACS Catal.* **11**, 11305-11319 (2021).

5 Valdés, Á., Qu, Z. W., Kroes, G. J., Rossmeisl, J., & Nørskov, J. K. Oxidation and photo-oxidation of water on TiO_2 surface. *J. Phys. Chem. C* **112**, 9872-9879 (2008).

6 Gono, P., Ambrosio, F. & Pasquarello, A. Effect of the solvent on the oxygen evolution reaction at the TiO_2–water interface. *J. Phys. Chem. C* **123**, 18467-18474 (2019).

7 Ping, Y., Nielsen, R. J. & Goddard III, W. A. The reaction mechanism with free energy barriers at constant potentials for the oxygen evolution reaction at the IrO_2 (110) surface. *J. Am. Chem. Soc.* **139**, 149-155 (2017).

8 Subbaraman, R. et al. Enhancing hydrogen evolution activity in water splitting by tailoring Li^+-$Ni(OH)_2$-Pt interfaces. *Science* **334**, 1256-1260 (2011).

9 Subbaraman, R. et al. Trends in activity for the water electrolyser reactions on 3d M (Ni, Co, Fe, Mn) hydr (oxy) oxide catalysts. *Nat. Mater.* **11**, 550-557 (2012).

10 Mathew, K., Sundararaman, R., Letchworth-Weaver, K., Arias, T. A. & Hennig, R. G. Implicit solvation model for density-functional study of nanocrystal surfaces and reaction pathways. *J. Chem. Phys.* **140**, 084106 (2014).

11 Mathew, K., Kolluru, V. S., Mula, S., Steinmann, S. N. & Hennig R. G. Implicit self-consistent electrolyte model in plane-wave density-functional theory. *J. Chem. Phys.* **151**, 234101 (2019).

12 Fernandez, D. P., Goodwin, A. R. H., Lemmon, E. W., Levelt Sengers, J. M. H. & Williams, R. C. A formulation for the static permittivity of water and steam at temperatures from 238 K to 873 K at pressures up to 1200 MPa, including derivatives and Debye–Hückel coefficients. *J. Phys. Chem. Ref. Data* **26**, 1125-1166 (1997).

13 Bi, Y. et al. Understanding the incorporating effect of Co^{2+}/Co^{3+} in NiFe-layered double hydroxide for electrocatalytic oxygen evolution reaction. *J. Catal.* **358**, 100-107 (2018).

14 Lee, Y. L., Gadre, M. J., Shao-Horn, Y. & Morgan, D. Ab initio GGA+ U study of oxygen evolution and oxygen reduction electrocatalysis on the (001) surfaces of lanthanum transition metal perovskites $LaBO_3$ (B= Cr, Mn, Fe, Co and Ni). *Phys. Chem. Chem. Phys.* **17**, 21643-21663 (2015).

15 Liu, D. D. *et al.* Heat-triggered ferri-to-paramagnetic transition accelerates redox couple-mediated electrocatalytic water oxidation. *Adv. Funct. Mater.* **32**, 2111234 (2022).

16 Tang, R. B. *et al.* Oxygen evolution reaction electrocatalysis on $SrIrO_3$ grown using molecular beam epitaxy. *J. Mater. Chem. A* **4**, 6831-6836 (2016).

17 Strickler, A. L., Higgins, D. & Jaramillo, T. F. Crystalline strontium iridate particle catalysts for enhanced oxygen evolution in acid. *ACS Appl. Energy Mater.* **2**, 5490-5498 (2019).

18 Chen, H. *et al.* Protonated iridate nanosheets with a highly active and stable layered perovskite framework for acidic oxygen evolution. *ACS Catal.* **12**, 8658-8666 (2022).
19 Shin, S. *et al.* Single-phase perovskite $SrIrO_3$ nanofibers as a highly efficient electrocatalyst for a pH-universal oxygen evolution reaction. *ACS Appl. Energy Mater.* **5**, 6146-6154 (2022).
20 Yu, J. *et al.* Monoclinic $SrIrO_3$: an easily synthesized conductive perovskite oxide with outstanding performance for overall water splitting in alkaline solution. *Chem. Mater.* **32**, 4509-4517 (2020).
21 Liu, Z. J., Wang, G. J., Guo, J. Y., Wang, S. Y. & Zang, S. Q. Sub-2 nm IrO_2/Ir nanoclusters with compressive strain and metal vacancies boost water oxidation in acid. *Nano Res.* **16**, 334-342 (2022).
